# Metal–Organic Framework-Based Materials for Wastewater Treatment: Superior Adsorbent Materials for the Removal of Hazardous Pollutants

**DOI:** 10.1021/acsomega.2c07719

**Published:** 2023-02-28

**Authors:** Harjot Kaur, Nishu Devi, Samarjeet Singh Siwal, Walaa F. Alsanie, Manju Kumari Thakur, Vijay Kumar Thakur

**Affiliations:** † Department of Chemistry, M.M. Engineering College, Maharishi Markandeshwar (Deemed to be University), Mullana-Ambala, Haryana 133207, India; ‡ Mechanics and Energy Laboratory, Department of Civil and Environmental Engineering, Northwestern University, 2145 Sheridan Road, Evanston, Illinois 60208, United States; § Department of Clinical Laboratories Sciences, The Faculty of Applied Medical Sciences, Taif University, P.O. Box 11099, Taif 21944, Saudi Arabia; ∥ Department of Chemistry, Government Degree College Sarkaghat, Himachal Pradesh University, Shimla 171005, India; ⊥ Biorefining and Advanced Materials Research Center, Scotland’s Rural College (SRUC), Kings Buildings, West Mains Road, Edinburgh EH9 3JG, United Kingdom; # School of Engineering, University of Petroleum & Energy Studies (UPES), Dehradun, Uttarakhand 248007, India; ∇ Centre for Research & Development, Chandigarh University, Mohali, Punjab 140413, India

## Abstract

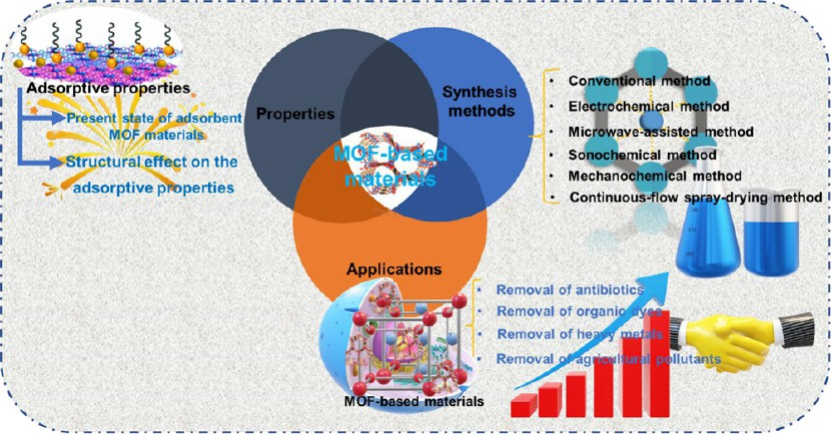

In previous years, different pollutants, for example, organic dyes, antibiotics, heavy metals, pharmaceuticals, and agricultural pollutants, have been of note to the water enterprise due to their insufficient reduction during standard water and wastewater processing methods. MOFs have been found to have potential toward wastewater management. This Review focused on the synthesis process (such as traditional, electrochemical, microwave, sonochemical, mechanochemical, and continuous-flow spray-drying method) of MOF materials. Moreover, the properties of the MOF materials have been discussed in detail. Further, MOF materials’ applications for wastewater treatment (such as the removal of antibiotics, organic dyes, heavy metal ions, and agricultural waste) have been discussed. Additionally, we have compared the performances of some typical MOFs-based materials with those of other commonly used materials. Finally, the study’s current challenges, future prospects, and outlook have been highlighted.

## 1 Introduction

Groundwater sources and external water decay by organic contaminants such as organic dyes, antibiotics, heavy metal ions, and pesticides have become a severe environmental issue. Manufactured dyes have a complex structure and are coloring agents that are nonbiodegradable with heightened resilience.^1,2^ Additionally, organic dyes are broadly utilized for dyeing a variety of products such as leather, textiles, medicine, and plastics.^3^ Fabric industries eliminate large quantities of polluted wastewater that possess organic dyes.^4^ Coloring reagents are one of the recognized types of water pollutants that should be extracted from wastewater prior to release within aquatic techniques.^5−8^ Actually, a concentration of 1.0 mg/L provides sufficient coloration and can be unsuitable for consumption by human beings and also contaminate water bodies. There are vast concerns related to the contamination of water by dyes as a kind of organic contaminant due to their persistence and coloration results.^9^ Antibiotics are widely utilized by humans as well as animal husbandry for the treatment of bacterial infections. More than one-half of antibiotic doses are excreted through urine from the body of human beings and animals because they cannot be adsorbed 100% and are mixed with water via flow streams. Many antibiotics are combined with water sources from hospital effluents, posing detrimental effects to the ecosystem.^10^ Antibiotics can cause several health problems, such as skin allergies, carcinogenic effects, and hereditary genetic defects, and can lower immune power.^11−13^

In addition, several industries such as civil construction, electroplating, metallurgy, and ceramics frequently produce heavy metal ions released to water resources via industrial wastewater.^14^ Heavy metal ions are toxic and have detrimental impacts on human health, as they can cause renal and neurological bioaccumulation and are poisonous to the digestive system.^15−17^ For farmland, pesticides are extensively used for better crop production, and eradication of these agricultural pests contaminates water sources and can damage the digestive, reproductive, endocrine, and nervous systems.^18^

The growing number of inhabitants and rise in water drinking have resulted in different processing techniques to remove contaminated components of anthropogenic sources within industrial wastewater before removing them to biological processes.^19−21^ Therefore, advancements in techniques and analyses used to develop photocatalytic composites have been of interest to numerous investigators. Industrial wastewater, such as fabric wastewater, is of primary consideration as this enterprise delivers enormous quantities of wastewater with an intense spectrum of chemical interpretations.^22^ In the coloring, printing, and finishing techniques, 10–50% failure of dyes from reactive pigments to the atmosphere is expected due to procedure inadequacy. Unfortunately, all of these contaminants cannot be removed entirely or degraded by traditional wastewater processing manufacturers within the atmosphere due to their continuous character in H_2_O and higher potency to rays, temperature, chemicals, and microbes-based invasion.^23−25^

MOF is a unique three-dimensional (3D) organic–inorganic composite with extremely porous nanostructures containing metal ions/groups and organic linkers.^26,27^ MOF has been carefully studied for several decades and has evolved as one of the magnetic materials for scientists and inventors because of promising components with tunable pore networks, adequate adsorption places, etc. The studies have also shown an in-depth investigation of MOF materials in previous decades, demonstrating their exceptional performance within catalysis,^28,29^ adsorption,^30,31^ and water harvesting.^32^ However, conventional catalysts and adsorbents such as activated carbons, clay minerals, zeolites, etc., have poor adsorption and pollutant removal capacity as compared to those of MOF-based NMs.^33−36^

Investigators have begun to manage the resilience of MOFs within diverse conditions, to comprehend the potential decay pathways, and to endeavor to create more durable framework networks.^37^ The strength of MOFs may be influenced by numerous aspects, such as the working atmosphere, metal ions, organic ligands, metal–ligand organization geometry, hydrophobicity of the aperture texture, etc.^38,39^ Also, synthesizing MOFs-based composites could help to improve the strength and performance toward practical applications. A review in this regard is provided by Ahmadijokani et al., where they explained the electrospinning synthesis route to synthesize MOF composites and hence their applications toward wastewater management.^40^ Investigations on the durability of MOFs have permitted us to explain the impact of some aspects and judiciously prepare sturdy framework networks. The somewhat labile coordination binds, which sustain the framework systems, are considered liable for the little resilience of MOFs.^41^ Therefore, a steady MOF system should have robust coordination binds to endure the invasion of guest molecules or have steric hindrances to control the intrusion of guests into the metal nodes.

Use of MOFs as support matrixes has been studied to combine different functional materials, for example, NPs,^42−44^ bioentities,^45^ and polymers,^46^ which offer rise to composites with improved or unique effects as related to their parental frameworks. Between these MOF composite substances, it may further decrease the size of the support MOFs and/or the active guest materials to nanoscale control to afford MOF composites.^47^ Combining different functional NPs, such as Au,^48^ quantum dots,^49^ and up conversion NPs,^50^ within MOF networks allows the nanocomposites to maintain the properties arising from the universal crystalline and porous configurations of MOFs and even to support the exceptional biomimetic catalytic, optical-electrical, and magnetic characteristic of the NPs. Similarly, the synergistic impact of integrating these materials may stimulate unique chemical and physical effects. The emergence of nanocomposites in this area should attract concentration to the potential possibilities and issues of these nanomaterials to reduce their progress in innovation and functionalities and donate to their growth potential utility for biomedical work. Up to now, NPs comprised into MOFs for catalytic utilizations have been broadly investigated and outlined.^51,52^

**Figure 1:**
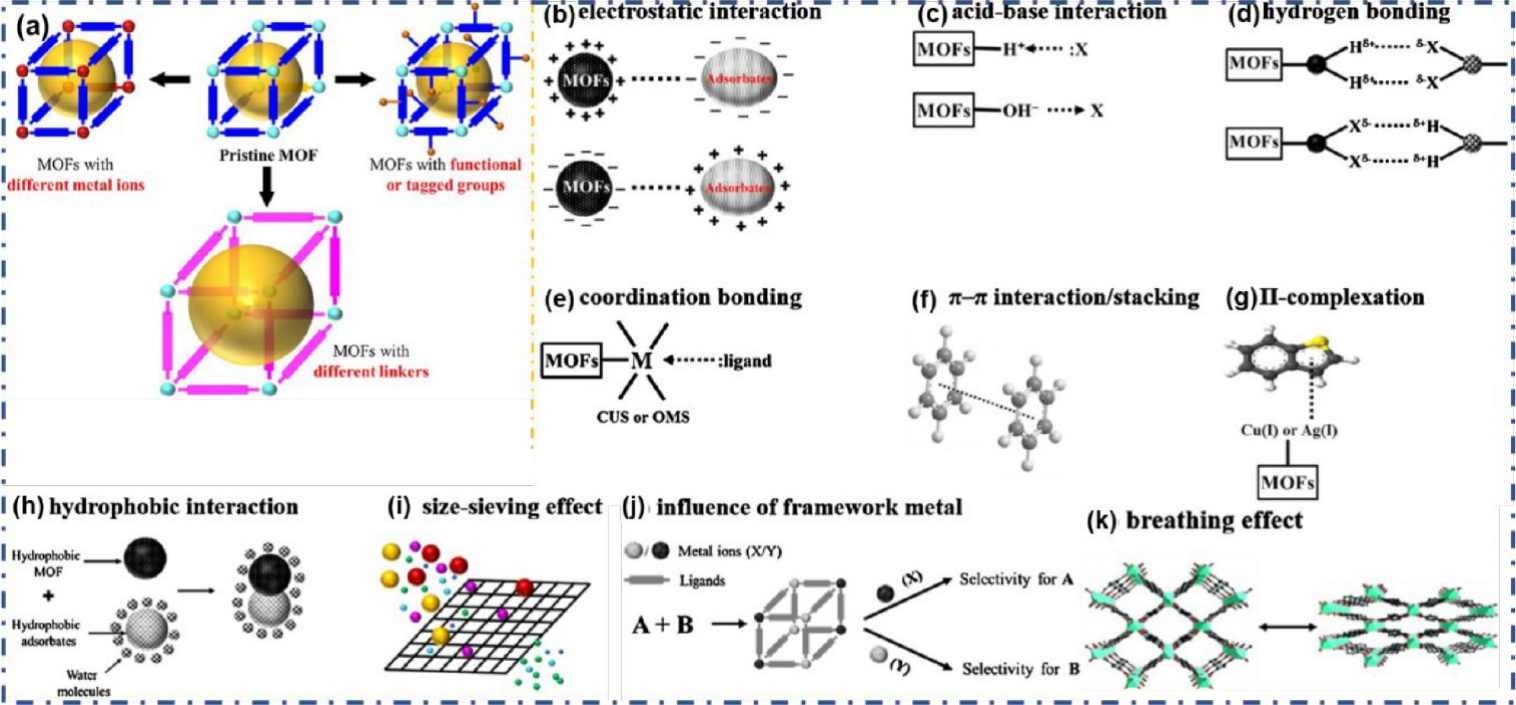
(a) Schematic representations of comparable MOFs. (b–k) Graphic expression of potential mechanisms for adsorption on the MOFs. Reprinted with permission from ref (53). Copyright 2021 Elsevier.

MOFs have drawn considerable concentration because of their increased porosity, designable pores, and easy preparation. Some fascinating MOFs are available, including isostructural, isoreticular, isomorphous, or similar types. As illustrated within Figure 1a, MOFs with identical crystal systems may be comprised of various metal ions (or groups) and linkers; thus, it may be convenient to examine the assistance of separately metallic components or linkers to adsorption, catalysis, and the band gap. Furthermore, MOFs can have functionality or FGs that profoundly impact the relations with adsorbates or supports (for catalysis). Figure 1b–k displays a rephrased plan for the noted tools associated with water and fuel sanctification through adsorption.^53^

This Review focused on the synthesis process (such as the traditional, electrochemical, microwave, sonochemical, mechanochemical, and continuous-flow spray-drying methods) of MOF materials. Moreover, the properties of the MOF nanomaterials have been discussed in detail. Further, MOF nanomaterials’ applications for wastewater treatment (such as the removal of antibiotics, organic dyes, heavy metal ions, and agricultural waste) have been discussed. Additionally, we have compared the performance of some typical MOFs-based nanomaterials with those of other commonly used materials. Finally, the study’s current challenges, future prospects, and outlook have been highlighted.

## 2 Synthesis of the Metal–Organic Framework

A growing field of analysis favorably complementary to the finding of unique MOF substances is related to the effect of fabricating and processing methods that permit the substances’ design to be used explicitly within all measurement hierarchies. These constructions are based on the molecular-scale methods that alter the chemical arrangements of substances and examine how to manage MOFs’ characteristics at high order ranges through the fine-tuning of networks.^54^ These treatment strategies are of high functional significance in allowing MOFs to be provided in arrangements that are amenable to combination into exact design compositions and are also anticipated to enhance the handling aspects of the substances while they are made in the industrial ranges.^55,56^

For any MOF approach to be industrially viable, several vital factors have to be assessed: (i) a universal approach is necessary to adjust the highest digit of MOF networks with an identical piece of tools; (ii) the opportunity to bypass complicated processing prerequisites such as high temperature and pressure will decrease capital and working expenses and ease protection circumstances; (iii) a control from set to continued processing could be helpful to deliver a higher outcome per unit time and a constant steady-state process that guides toward seriously decreased rests, work expenses, reactor volumes, and constant and uniform exhibit; and (iv) an elevated space-time-yield (STY) parameter that estimates the quantity of MOF constructed per m^3^ reaction combination per day. Nevertheless, these techniques are such that they would be generalized to any MOF design, delivered to properly optimize the reaction or fabrication essentials. Similarly, it can be anticipated that the application of MOF superstructures on a large scale will inevitably be plagued by economic and ecological concerns of their preparation systems.

**Figure 2:**
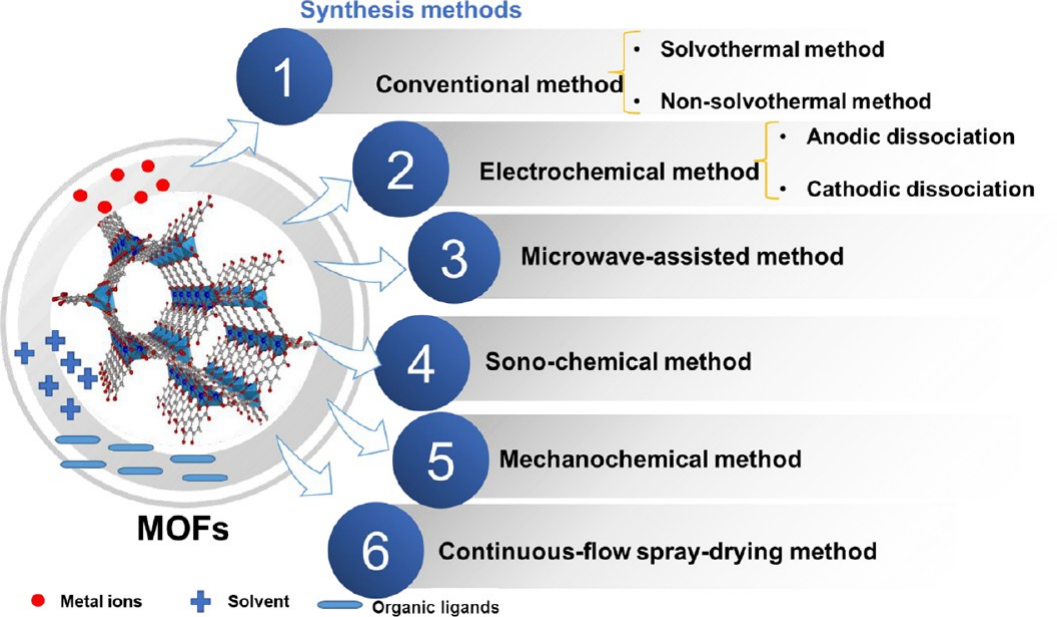
Synthesis process of MOFs.

Since the discovery of MOFs, several methods such as electrochemical, microwave, sonochemical, and solvothermal have been developed to synthesize MOFs. Unreacted molecules, organic, and inorganic molecules play a pivotal role in the formation of MOFs. Two main steps that are important after the synthesis of MOFs are purification and activation,^57^ as impurities can reduce the adsorption ability and performance of the material. Sometimes heating is required for the activation of MOF materials, which causes the collapse of the MOF network with guest molecules. So, to simplify the activation process, we can utilize different methods, such as replacing the nonvolatile with a volatile solvent, to lessen the required deactivation temperature. In this section, we briefly describe different methods to synthesize MOFs (Table 1). Figure 2 shows the synthesis process of MOFs.

**Table 1:** Summary of the Synthesis Methods with Encountered Challenges

synthesis method	pros	cons	challenge
conventional	easy and simple	time and temperature regulation difficult	controlling morphology and degradation
	no need of mechanical equipment	not suitable for large-scale applications	
electrochemical	ambient conditions	energy use	electrode selection for practical applications
	controlling film	uniform film thickness	
microwave-assisted	accelerated nucleation and growth rate	need pairing of other methods for better results	need special setup and design of equipment
	easy pairing with other methods		
sonochemical	enhance crystallization and growth	detrimental effects of ultrasonic waves	large-scale design and application
	quick and consistent nucleation		
mechanochemical	green approach	morphology control	controlling nucleation and crystallization without solvent
	ambient condition		
	solvent-free approach		
continuous full spray drying	one step	design flexibility	structure and morphology control
	industrial		

### 2.1 Conventional Method

Solvothermal and nonsolvothermal methods are included in conventional methods. In the solvothermal method, MOFs are developed in sealed nuclear magnetic resonance (NMR) tubes or in vials through conventional electric heating in small intervals.^58^ The term “solvo” indicates the solvents, such as ethanol, methanol, formamides, acetones, and water. This method is used by Huang et al.^59^ to fabricate Cu_3_(benzene tricarboxylic acid)_2_ (Cu_3_(BTC)_2_) and Cu(benzene dicarboxylic acid) (Cu(BDC)) MOFs for the treatment of phenol wastewater. Underneath the identical circumstances, the Cu(BDC) exhibited a higher catalytic performance as compared to that of the Cu_3_(BTC)_2_, which was primarily ascribed to the unique design of the Cu(BDC), directing accessible entry within the holes for the organics. The prepared Cu(BDC) showed a solid capability to adjust to the imitation phenol wastewater of distinct concentrations. Thus, Cu-MOFs would promise heterogeneous catalysts for the catalytic wet peroxide oxidation of organic effluents. Two types of mixtures were prepared, first with 1.94 g of Cu^2+^ nitrate trihydrate mixed in 30 mL of deionized water, and second with 0.84 g of 1,3,5-benzenetricarboxylic mixed in 15 mL of ethanol and dimethylformamide (DMF) each. Both of the solutions are mixed together with stirring until the suspension becomes homogeneous. The obtained solution was then transferred to an autoclave that was sealed and heated to 110 °C. Deep blue crystals were obtained after the reaction was cooled at room temperature. The authors observed a phenol conversion efficiency of 99%. Amino-functionalized Ti(IV)-based MOFs were fabricated by Wang et al.^60^ for the removal of Cr(VI) from wastewater via the solvothermal technique. The material possessed a higher specific surface area of 1343.9 m^2^ g^–1^. The microwave-assisted solvothermal method was utilized by Nguyen et al.^61^ for the development of bimetallic metal (Ni, Mg, and Sn)/MOFs, which showed 96% removal of rhodamine B, crystal violet from the wastewater.

Despite numerous benefits, time and temperature must be regulated tightly in the solvothermal approach. For instance, the difference in temperature can influence the particle morphology, and the reaction time extension may direct MOF degradation.^62,63^

Nonsolvothermal techniques are simpler than solvothermal for the synthesis of MOFs. Mechanical, nanoprecipitation, and emulsion are the methods that are involved in a nonsolvothermal approach for the fast growth of MOFs.^64^ In this strategy, complex equipment is not required; below the boiling point temperature of the solvent, and at atmospheric pressure, MOFs can be fabricated in an open vessel. Modification of pH and temperature is concerned with obtaining the maximum yield of the MOF material. For instance, to synthesize MOF-74 (Zn), Zhang et al.^65^ carried out an adapted method without the utilization of microwave/ultrasonic treatment, and no extra pressure was provided to the system. In brief, 239 mg of 2,5-dihydroxy terephthalic acid and 686 mg of Zn (OAc)_2_·2H_2_O were dissolved in dimethylformamide (20 mL). The obtained solution was then added to the salt solution with constant stirring for 18 h at room temperature to produce MOF-74 (Zn). The MOF-74 (Zn) was dried in air and evacuated at 270 °C. Finally, MOF-74 (Zn) was added to 1.14 mL of ethylenediamine and toluene solution, which was dried in air to yield ammoniated MOF-74 (Zn). Ammoniated MOF-74(Zn) byproducts are a luminescent sensor for selective tetrabromobisphenol A (TBBPA) detection. The fluorescence enhancement delivered an excellent linear association with the concentrations of TBBPA in the capacity of 50–400 μg/L, and its detection limit could reach 0.75 μg/L.

However, traditional methods of MOFs synthesis yield a fine powder, so they are not commercially applicable to much extent.

### 2.2 Electrochemical Method

An electrochemical preparation process is a standard approach that permits monitoring the film deposit operation via modifications within the involved voltage or current to prevent film consistency. This is a technique with a quick reaction rate and gentle circumstances. It may be brought out under ambient conditions, and the valuable equipment is somewhat easy.^66−68^ Currently, electrochemical preparation approaches may be separated within the anodic electrodeposition,^69^ cathodic electrodeposition,^70^ and electrophoretic deposition techniques.^71^

MOFs can be synthesized and deposited directly as well as indirectly. Required MOFs are generated directly on the surface of the electrode via electrochemical reactions in the former, while in the latter, an electrochemical reaction is a step of the procedure to synthesize MOFs. Electrochemical methods can be of two types such as anodic dissolution and cathodic electrosynthesis.

#### 2.2.1 Anodic Dissolution

In this method, a potential or current is applied to the electrode, which is dipped inside the solution consisting of an organic ligand and electrolyte. After anodic voltage is applied, oxidation of metal takes place to form metal ions and liberated into the solution of organic linkers. A thin layer of MOF is formed on the surface of the electrode due to the reaction of metal ions and organic ligands.^72^

#### 2.2.2 Cathodic Dissolution

In this system, when a specific voltage is applied to a metal precursor, organic ligand, and pro-base-containing solution in an appropriate electrolyte, MOFs are formed on the cathodic electrode.^67^

**Figure 3:**
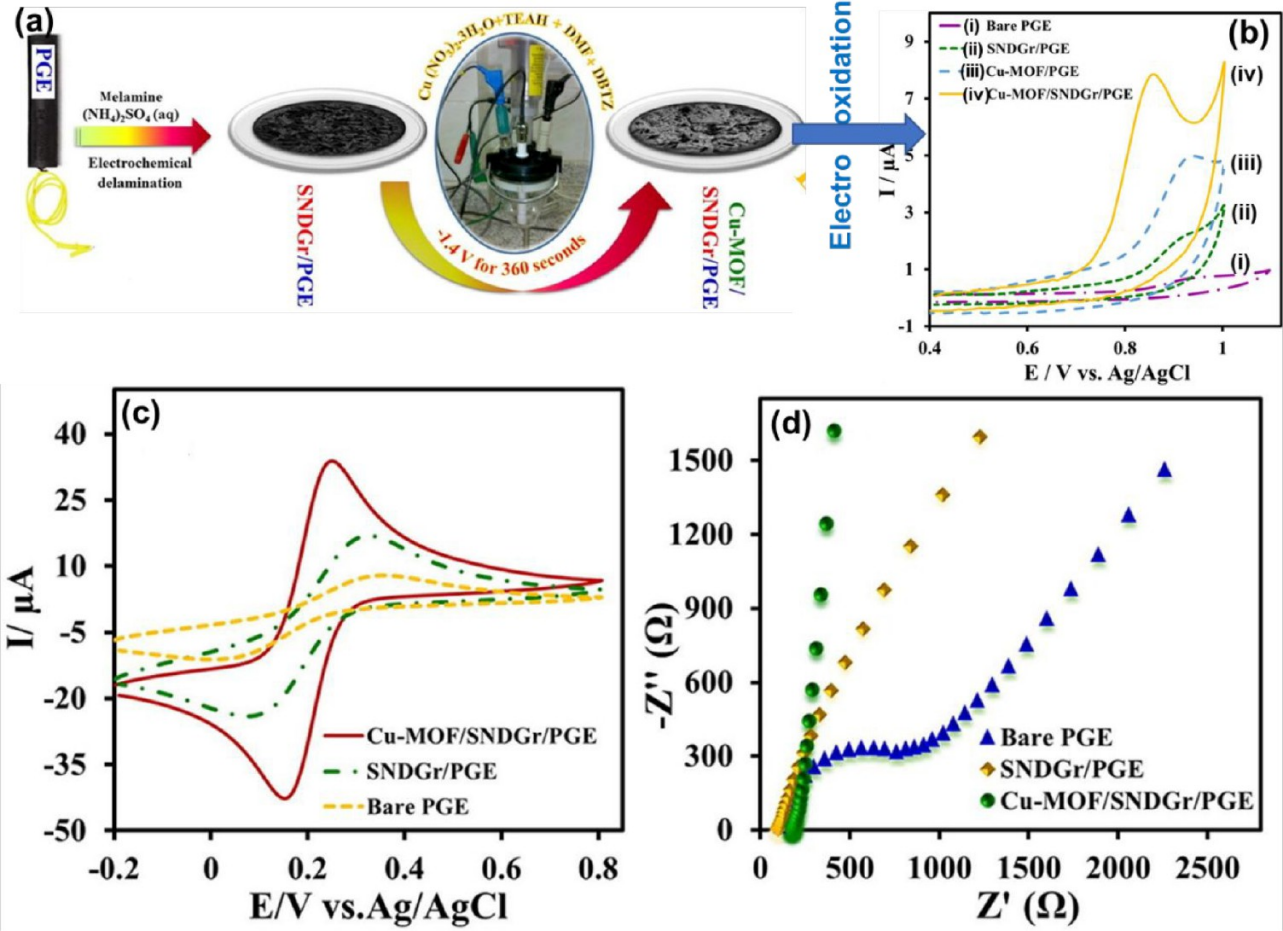
(a) Schematic illustration of the Cu-MOF/SNDGr/PGE and its application. (b) CVs at a sweep speed of 50 mV s^–1^ of different synthesized material (0.1 M, pH = 7.0) solutions comprising 11.8 μM STLHC. (c) CVs of the pristine PGE (i), SNDGr/PGE (ii), Cu-MOF/PGE (iii), and Cu-MOF/SNDGr/PGE (iv) in 0.1 M KCl comprising 5 mM [Fe(CN)_6_]^3–/4–^. (d) Nyquist spectra of pristine PGE, SNDGr/PGE, and Cu-MOF/SNDGr/PGE with the identical media. Reprinted with permission from ref (73). Copyright 2021 Elsevier.

Some of the examples of MOFs synthesis via electrochemical methods are described next. Habibi et al.^73^ fabricated Cu-based MOF on S, N-doped graphene nanocomposite (SNDGr) via an electrochemical technique as showcased in Figure 3a for the detection of sertraline hydrochloride. Briefly, for the Cu^2+^ source, Cu(NO_3_)·3H_2_O (0.577 g) and triethylamine hydrochloride (0.12 g) as a supporting electrolyte were utilized and dissolved in 5 mL of dimethylformamide (DMF). This solution was considered the first solution. After that, disodium 5,5′-bitetrazole-1-ide(Na_2_C_2_N_8_) was dissolved in 5 mL of DMF. This solution was then added to the first solution with continuous stirring for an early 2 h, and the resulting mixture was sonicated for 5 min. Afterward, SNDGr/pencil graphite electrode (PGE) was dipped into the obtained solution, and a cathodic current of −1.4 V was applied, resulting in the formation of Cu-MOF/SNDGr/PGE. Considerable enhancement within the oxidative peak current at a lower voltage toward electrooxidation of sertraline hydrochloride (STLHC) upon the Cu-MOF/SNDGr/PGE would signal that, for quantitative electrochemical finding STLHC, a prudent sensor could be made. Eventually, on the reverse scan, as shown within Figure 3b, no reduction peak occurred, indicating the irreversible nature of the electrochemical method of the STLHC upon the all-incorporated electrodes. For electrochemical analyses, identification of the electrochemical active surface area (ECSA) is essential. The electrocatalytic description of various electrodes was studied through the cyclic voltammetry (CV) approach utilizing the typical redox electrode design, [Fe(CN)_6_]^3–^/[Fe(CN)_6_]^4–^, into 0.1 M KCl medium. A pair of finally described peaks of the CVs curves of the probes is showcased in Figure 3c. The acquired CVs indicate that the peak voltage split (Δ*Ε*_p_) of Cu-MOF/SNDGr/PGE, SNDGr/PGE, and pristine PGE is 0.106, 0.171, and 0.251 V, respectively. The reductive and oxidative peak currents of Cu-MOF/SNDGr/PGE are 2- and 4-fold more elevated in comparison to the SNDGr/PGE and bare PGE concurrently. The interfacial characteristics of the probe and solution were examined by the electrochemical impedance spectroscopy (EIS) process, which was conducted within 0.1 M KCl media via utilizing the standard [Fe(CN)_6_]^3–^/[Fe(CN)_6_]^4–^ redox method as the electrode. At an open circuit voltage within 1 Hz to 100 kHz, the Nyquist plots for the pristine and doped PGEs are displayed in Figure 3d. The Nyquist plot of EIS at higher frequencies comprises very short semicircles associated with the electron transfer procedure within the interface of the electrode/solution, and at lower frequencies, a direct line associated with the scattering method is shown.

Zhou et al.^74^ electrochemically synthesized a series of face-centered cubic MOFs, which efficiently decreased the energy input by approximately 90% as compared to the single distillation process, a conventional method. Overall, the electrochemical process may be involved beneath favorable processing circumstances and constantly nucleate at all temperatures, and it also permits surface changes.

### 2.3 Microwave-Assisted Method

Microwave treatment had a shorter reaction time for the synthesis of MOFs, which was reported in 2005 for the first time. The authors observed that a reduced time of synthesis did not affect the yield much as 44% material was obtained in 4 h in the case of the microwave-assisted method, while in the case of a traditional heated oven 45% yield was obtained after 4 days. A reduction in synthesis times was progressively obtained since 2005.^75^ Further, narrow sized crystals or reduced particle size MOFs can be obtained via microwave-assisted techniques in comparison to conventional ovens, and even the size of the crystal can be controlled during synthesis.^76^ Lev Bromberg et al.^77^ elucidated that MIL-101 synthesized via the microwave-assisted method exhibited a larger surface area (4004 m^2^/g) as compared to autoclave-synthesized MIL-101, which had a shorter surface area of 3460 m^2^/g. Therefore, a larger surface area, controllable particle size, and a shorter time for reaction are the significance of microwave-assisted methods for the fabrication of MOF-based materials.

Microwave-assisted syntheses of MOF have been widely used. In a Teflon vessel, a mixture of substrate and the appropriate solvent is taken, and then the vessel is sealed and kept inside a microwave unit at a certain temperature for a set time.^78^ Ren et al.^79^ designed Zn-MOFs for the effective degradation of contaminants such as tetracycline hydrochloride and Congo red from wastewater by the microwave-assisted ball-milling technique. Another team of researchers fabricated NH_2_-MIL-125(Ti) MOF using the microwave-assisted method for the removal of diclofenac. Here, MIL refers to Materials of Institute Lavoisier, a kind of MOF where the Ti_8_O_8_(OH)_4_ metal cluster is linked with six benzene-1,4-dicarboxylate units.^80^ MOFs were synthesized at 200 °C under microwave radiation for 15 min and showed a surface area of 1030 m^2^/g and a pore volume of 0.45 cm^3^/g, which indicates the developed porosity. In the batch test, diclofenac was completely removed within 3 h.

Electrochemical preparation and microwave radiation were suggested to deposit a patterned luminescent MOF (LMOF) upon conductive glass.^81^ Mainly, a film of lanthanum hydroxide is incorporated upon a fluorine-doped tin dioxide (FTO) exterior via electrochemical deposition. Afterward, beneath microwave irradiation, the hydroxide film was altered to LMOF. The microwave-aided preparation approach may significantly accelerate the MOF particles’ nucleation and evolution rate due to its rapid heating, thereby obtaining MOF substances with smaller sizes and more fair distribution that may be involved in catalysis. This method is suitable for most lanthanide ions and has effective energy transferability. Furthermore, they have potential application in the field of anticounterfeiting barcodes.

### 2.4 Sonochemical Method

**Figure 4:**
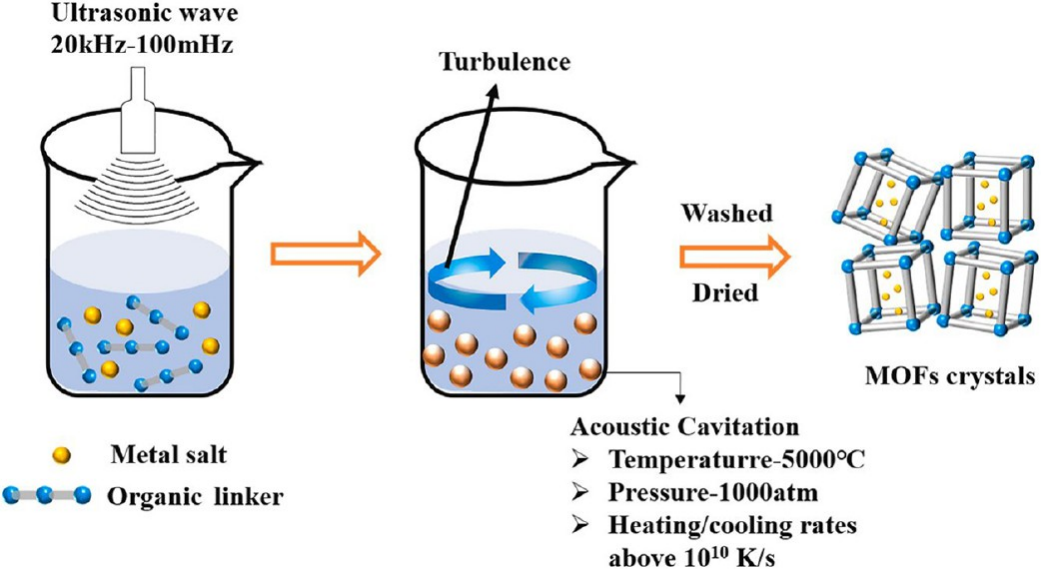
Graphical representation of the synthesis of MOFs by the sonochemical method. Reprinted with permission from ref (85). Copyright 2021 Elsevier.

Ultrasonic rays provide energy to the reaction between the metal ion origin and the organic ligand. The sonochemical approach may enhance the crystallization and development speed during the growth procedure.^82,83^ ZIF-8 layers may be prepared through a sonochemical approach. Initially, Zn(NO_3_)_2_·6H_2_O and 2-MeImi were liquefied within DMF and mixed continuously until a clear solution was received. After that, TEA (triethylamine) was integrated within the solution, and the solution was fast assigned to the reactor and put upon the ultrasound rod. The ZIF-8 specimen was rinsed with DMF and absorbed into methanol. After filtration, the specimen was parched. The sonochemical process facilitates quick and consistent nucleation, and the outcome of ZIF-8 acquired through this process is very high.^84^ The synthesis of MOFs using the sonochemical technique is shown in Figure 4.^85^

The reactions between organic ligand and metal ion sources are initiated by ultrasonic radiation in the case of the sonochemical synthesis of MOFs. Under mild conditions, it can improve the surface morphology and growth rate and crystallization.^86^ MOF-177 was synthesized by Jung et al.^83^ by utilizing 1-methyl-2-pyrrolidinone as a solvent via the sonochemical technique with a Pyrex reactor attached to a sonicator. The fabrication was conducted in He with no usage of external cooling. Crystals were obtained within 40 min and stored for 3 days in dichloromethane, and then vacuum-dried for 1 day. The surface area of the obtained MOF was in the range of 4200–4900 m^2^/g, and the product yield was 95.6%. However, the desire for green synthetic methods is increasing daily to minimize the detrimental impacts on the ecological system. Thus, we also discuss some green synthesis for the preparation of MOFs.

### 2.5 Mechanochemical Method

**Figure 5:**
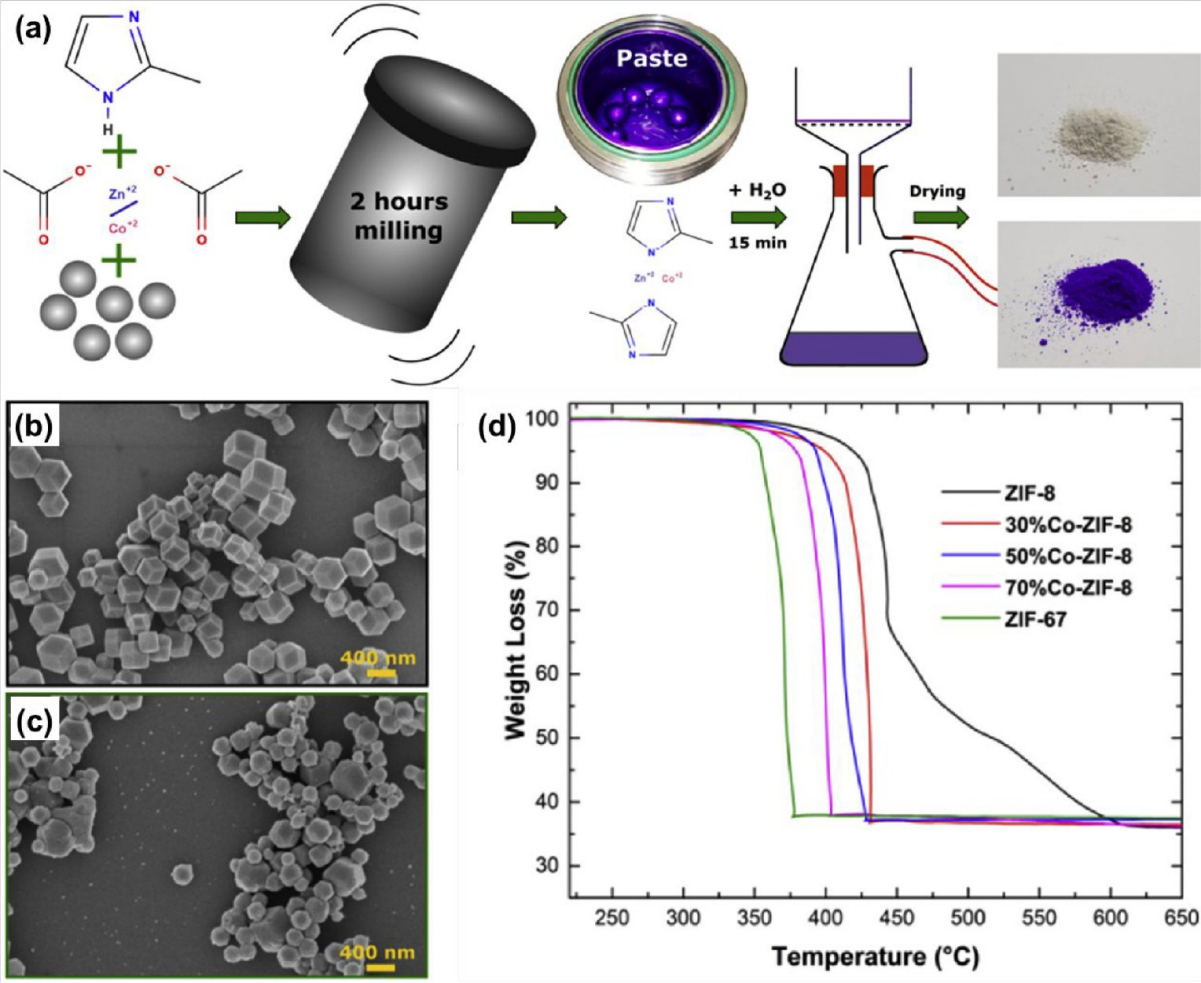
(a) Representation of the synthetic process of Co-containing ZIF-8; (b,c) SEM pictures of ZIF-8 and ZIF-67; and (d) thermogravimetric analysis of the synthesized materials. Reprinted with permission from ref (89). Copyright 2020 Elsevier.

Mechanochemical synthesis gained more attention as a greener method than did solution-based routes as the energy is directly transferred between solid-phase reactants, and a minimal amount of solvent (or solvent-free conditions) is required to assist the mechanochemical reactions known as liquid-assisted grinding. In addition, these reactions can generally take place at room temperature and are less time-consuming.^87^ Typically, ball-milling/grinding of the mixture of solid precursors in a ball-miller is involved in the mechanochemical synthesis, which provides an opportunity to utilize insoluble metal sources.^88^ Zeolitic imidazole frameworks ZIF-8, Co-containing ZIF-8, and ZIF-67 were synthesized by Taheri et al.^89^ where the authors found that ZIF-8 is more appropriate for water-related applications with a surface area of 1881 m^2^/g as compared to ZIF-76, which had a surface area of 1525 m^2^/g. Ball-milling was utilized for 2 h to fabricate MOF-based materials. Figure 5a illustrates the synthesis process of Co-containing ZIF-8, Figure 5b,c represents the SEM images of ZIF-8 and ZIF-67, and Figure 5d shows the thermogravimetric analysis from 300 to 650 °C with a weight loss of nearly 63%, which confirms the decomposition of ZIF to metal oxides. The selection of suitable MOFs for water treatment was directed in this study. Various zirconium-based MOFs are synthesized using the mechanochemical technique. For instance, the UiO-66 analogue was fabricated via a mechanochemical approach without acidic additives using a liquid-assisted grinding process with water.^90^ This provides new paths for converting biomolecules into MOFs that can be used as composites in numerous applications.

### 2.6 Continuous-Flow Spray-Drying Method

For the production of MOFs on a large scale for the industrial exploitation and continuous synthesis of MOFs in the form of NPs, composites, and spherical structures, the continuous-flow spray-drying technique attracted much attention due to its cost-effectiveness and environmentally friendly process.^91^ This technique is a combination of both spray-drying and continuous-flow techniques. Several members of the MOFs family involving UiO-66 and Fe-BTC/MIL-100 were synthesized by Garzón-Tovar et al.^91^ Briefly, they introduced a continuous-flow reactor at the nozzle of the spray dryer. Initially, in a continuous flow reactor, the precursor solution was injected, which contains metal salt and organic linker, and it was heated to a temperature that promotes nucleation. The outlet of the flow reactor was directly connected to the entrance of the spray dryer; the solution was automatically injected into the spray dryer. Here, the growth of the MOFs was confined to the atomized droplets and collected as micro spherical beads. In addition, the utilized solvent could be recovered, making the process cost-effective and waste-efficient. UiO-66-NH_2_ and Zr-fumarate beads were synthesized by Avci-Camur et al.^92^ Briefly, a mixture of 2-aminoterephthalic acid with H_2_O and CH_3_COOH in an equimolar ratio with ZrOCl_2_·8H_2_O was inserted into the coil-flow reactor and placed in a silicone bath. The resulting yellow-colored slurry was spray-dried, and the beads were collected and dried at 75 °C. The surface area of UiO-66-NH_2_ was 840 m^2^/g. This synthesis method is integrated as a green approach in many industrial sectors for the continuous one-step preparation of MOF beads.

## 3 Properties of the Metal–Organic Frameworks

**Figure 6:**
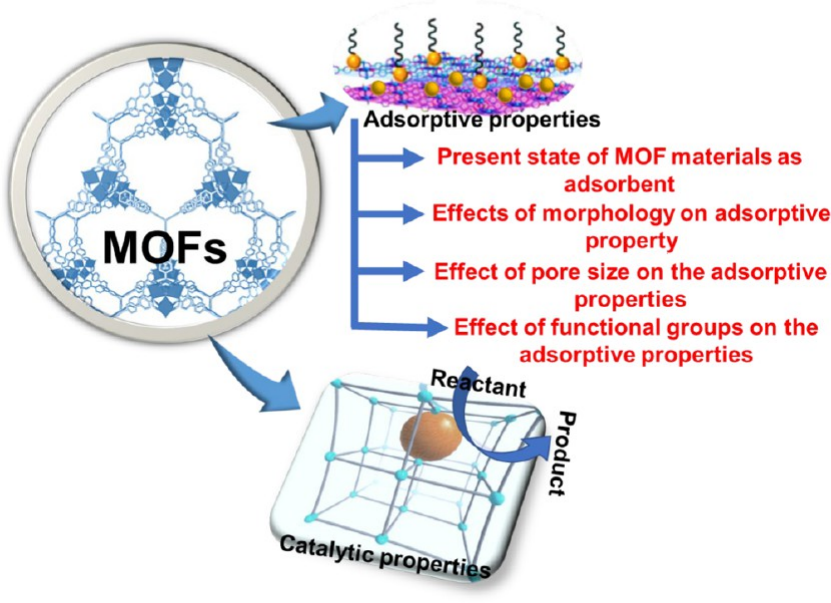
Properties of the MOFs materials.

The adsorptive and catalytic properties of the MOF-based materials have great potential in drug delivery,^93,94^ luminescence,^95,96^ sensing and degradation,^97,98^ and in the removal of toxic pollutants from wastewater. They possess tunable pore sizes, outstanding thermo-chemical stability, and large amounts of surface area. We briefly explain the adsorptive and catalytic properties of MOF-based materials in separation, sensing, and environmental remediation. Figure 6 shows the proposed properties of the MOFs.

### 3.1 Adsorptive Properties

Adsorptive elimination of poisonous contaminants from wastewater is considered to be one of the most challenging and outstanding research areas toward the protection of the environment and management of water due to cost-effectiveness, user and eco-friendly synthesis, facile operation, and recyclability. Unsaturated metal sites and charge on the MOF-based composites play a significant role in dye adsorption and removal properties as these active adsorption sites of MOF can interact supermolecularly with the molecules of dyes.^99^ Generally, the mitigation or elimination of toxic molecules from aqueous solutions via adsorption requires a known amount of MOF-based material in polluted aqueous solution for the particular time period, so that pollutant molecules get adsorbed on the surface of MOF, which ultimately can be removed by mechanical methods. Therefore, thermodynamic water stability is an important factor in adsorption.

#### 3.1.1 Present State of MOF Materials as Adsorbent

Decades of essential investigation on reticular chemistry have afforded universal MOF systems and allowed a precise acquaintance of their effects. This has paved the path for a paradigm change from basic science to the advanced investigation. While previous years have seen a considerable improvement in the chemistry and application of MOFs, there remains ample space for study, especially in the context of commercialization. Concerning the emerging area of MOFs, more steps must be dedicated to the fundamental investigation into their properties to fully employ their potential in applied analysis. In contrast, using the extensive body of actual study on MOFs, placing and optimizing substances for typical utilizations, and pushing reticular materials from the lab to industrial applications is essential.^100^

As discussed in the previous sections, the adsorbents’ porosity, pore geometry, and distinct adsorption locations are crucial in efficient adsorptive reduction. The pores of solids can be classified on the basis of their sizes.^101^ Conventionally, the topography of adsorbents has been overwhelmed via substances, for example, zeolites, activated carbon, silica, waxes, and alumina. These substances propose a generous concession among price, ability, and selectivity. The majority of aluminosilicate zeolite, untreated alumina, and activated carbon (AC) substances are hydrophilic and absorb from organic media. In contrast, AC is hydrophobic and thus competently works to absorb organic contaminants from water. Therefore, AC is presently the considerable standard adsorbent substance for wastewater processing.

A recent study on adsorption substances primarily concentrates on producing and optimizing specific adsorbents to utilize them for application. Other agricultural, municipal waste, and industrial rivulets have been supposed to produce low-price adsorbents. In contrast, adsorbents with extraordinary capability or selectivity have also been developed. MOFs are classified as engineering materials with porous organic networks that make them unique with extraordinary performance as nonconventional adsorbents.

#### 3.1.2 Effects of Morphology on the Adsorptive Property

**Figure 7:**
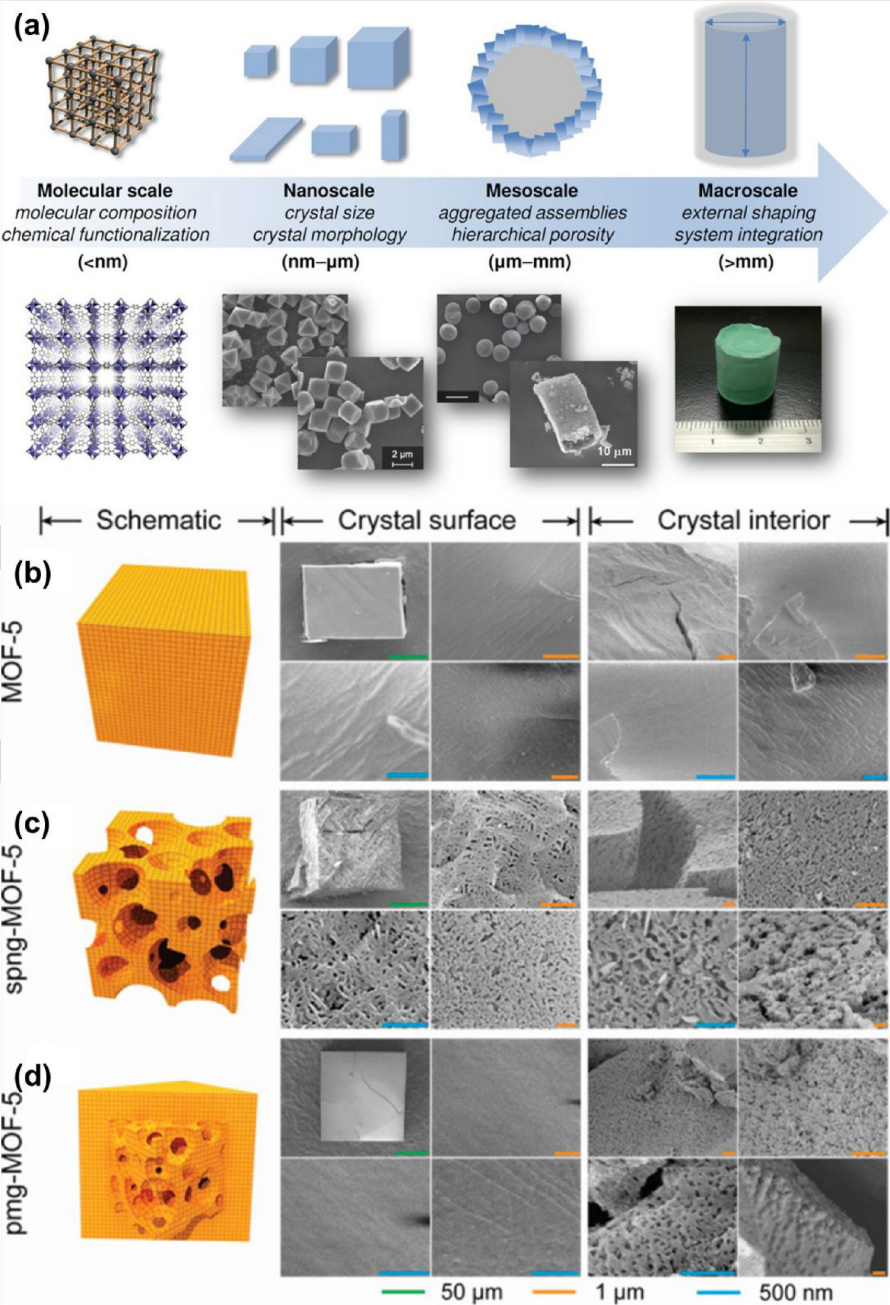
Measurement ranges applicable to the optimization of MOFs (a). Schematic graphs and scanning electron microscopy (SEM) pictures of the crystal interior as well as the exterior for (b) cubic, (c) spng, and (d) pmg-like crystals of MOF-5. Reprinted with permission from ref (102). Copyright 2018 John Wiley and Sons.

It is clearly understood that the structural morphology and orientation could significantly impact the performance of MOFs as adsorptive materials. In regard to this, Oliver et al.^102^ showed the effect of high order structurization on the adsorptive characteristics of MOFs. Significantly, from an essential viewpoint, the high order structurization of MOFs delivers the option for unique effects to occur that are self-sustaining of the molecular arrangement and system configuration of the MOF. The measurement scales appropriate to MOFs’ optimization are shown in Figure 7a. In the molecular range, the modular manufactured process authorizes the metal and organic ingredients to be rationally chosen, such that their positions are comprised into the resultant framework. It permits parameters, for example, the chemical functionalities and pore dimensions, to be finely adjusted according to the chosen substance characteristics. On the nanoscale, the characteristics of particular crystals are managed, for example, crystal dimensions and morphology that may show benefits, such as the adsorption kinetics. Individual crystals may be employed as assembling unions to make big groups at the mesoscale, directing refined architectures, for example, hollow spheres, thin layers, and monolithic designs. Ultimately, structuralization on the macroscale delivers ways to shape the MOF designs into the preferred structure. These scales provide many fascinating possibilities to increase the adsorptive effects of MOF techniques for the mesoscale and nanoscale structuralization of MOF.

However, in the cubic crystals brought within traditional syntheses (Figure 7b), higher amounts of 4-(dodecyloxy) benzoic acid (DBA) were delivered into a sponge-type (spng) crystal with pores that reproduced via the whole crystal (Figure 7c), while a “pomegranate” (pmg) surface, in which a thick texture retained a hierarchically porous network, was kept at low concentrations of the additive (Figure 7d). An uptake shape was shown by the adsorption isotherm of N_2_ toward the two hierarchically porous structures compatible with the high density of micropores originating from the MOF-5 network class. The N_2_ adsorption isotherms toward the two hierarchically porous structures showed an uptake shape compatible with a high density of micropores originating from the MOF-5 network class. However, a noticeable impact was not shown by mesopores as the supported macro- and mesopores even at high heat would have to be very large for their influence to be exerted on the adsorption profile of N_2_.

Solids with a high porosity, large surface area, and high metal ion densities are desirable for catalysis. Numerous MOFs have a surface area of about 5000 m^2^/g or more,^103^ and the presence of metallic nodes brings out the catalytic performance for several reactions. One of the reasons MOFs are more advantageous than other solid catalysts is the probability of structure and pore dimension prediction by knowing the structure of the linker and directionality of coordination among the metal clusters. For example, by replacing one linker with another having increased dimensions but the same geometry of carboxylate units, reticular MOFs can be obtained.^104^ MIL-101(Cr) is frequently used as an acid catalyst in aqueous solutions. It comprises two zeotypic mesopores of 29 and 34 Å diameter and a surface area of 4100 m^2^/g and is highly stable in catalytic reactions.^105^

**Figure 8:**
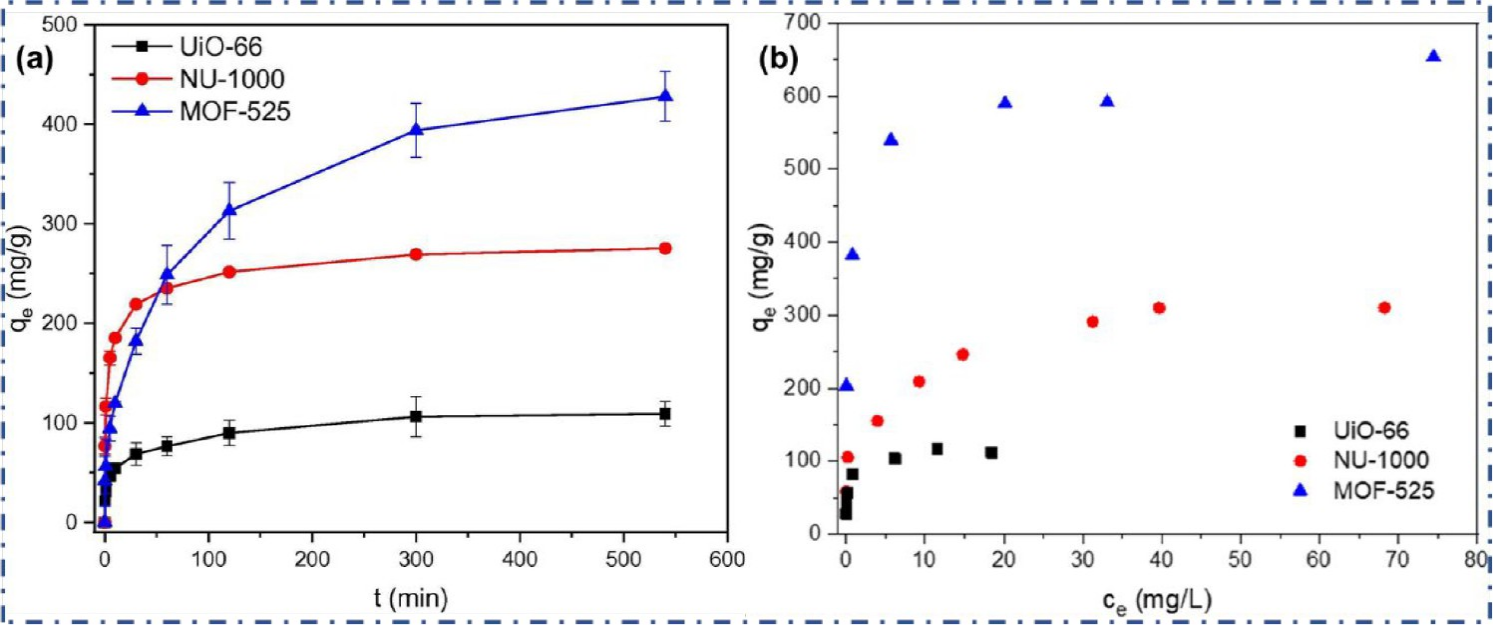
(a) Adsorption of TC on UiO-66, NU-1000, and MOF-525 with time and (b) adsorption isotherms of TC on as-synthesized MOFs. Reprinted with permission from ref (110). Copyright 2021 Elsevier.

The clusters of the framework act as nanosized oxides, and the surrounding linkers work as antennae.^106^ MOFs with transition metals such as Co, Cr, and Fe act as oxidation catalysts.^107^ MOF provides different active sites such as metal nodes with exchangeable coordination positions that are unconnected to the linkers, modified ligands with attached active sites, or inclusion within the void volume of the active species utilized as oxidation catalysts. Depending on the type of oxidation, these catalysts attain a different level of efficiency.^108^ Liu et al.^109^ fabricated Zr-based MOFs (UiO-66, NU-1000, and MOF-525) and reported adsorption isotherms corresponding to each morphology. Adsorption kinetics and isotherms are affected significantly by the different morphologies of the material. For instance, an adsorption equilibrium was reached in 120 min in the case of MOF-525, while in the cases of UiO-66 and NU-1000, it takes only 40 min to reach the adsorption equilibrium as described in Figure 8a. Sips model of adsorption isotherm was most suitable for the adsorption of TC on MOFs as showcased in Figure 8b. The adsorption capacity of TC was maximum for MOF-525 among the three prepared Zr-based MOFs, which is even higher than for other adsorbents, that is, 9.5, 3, and 5.4 times higher than those of granular AC, MWCNTs, and GO, respectively.

#### 3.1.3 Effect of Pore Size on the Adsorptive Properties

The pore size and geometry of the MOF-based materials are crucial for the adsorptive removal of dyes. Because of their higher surface area, porous MOFs can absorb more significant quantities of dyes than can nonporous MOFs. Flexible or adjustable design results in several MOF frameworks with multiple pore sizes, dimensions, and geometries. An exhaustive review by Cai et al.^27^ provides insights into this aspect, where they considered composites and MOF derivatives to explain the effects of mesopores on the microporous structures. For instance, MOFs such as IRMOF-01 and PCN-222 have square grid channels, while PCN-224 and MOF-74 have hexagonal channels.^111,112^ If the pore size of the MOF-based composites is larger than the size of the dye molecules, then a considerable amount of dye can be adsorbed on the surface of the MOF. However, if the size of the molecules of dyes is more significant as compared to the MOF frameworks, then the adsorption of dye molecules is ruled out within the pores of MOF.^113^ Also, Cui et al.^114^ reported the regime upon the pore chemistry and size within metal coordination systems with hexafluoro silicate and organic linkers toward preferential binding, as well as the tidy group of acetylene molecules via combined host–guest and guest–guest relations. The significance of these binding relations affords high adsorption ability and selectivity for acetylene at room temperature. Experimental breakthrough curves exhibit their efficiency in separating acetylene/ethylene combinations.

#### 3.1.4 Effect of Functional Groups on the Adsorptive Properties

The adherence of adsorbates is promoted by particular functional groups present in MOF-based materials, such as amino groups interacting with acidic dyes and sulfonic acid interacting with basic dyes and pore geometries of porous MOFs.^99^

Dyes can facilely be degraded via the oxidation process. The Fenton advanced oxidation process is considered a potential method for removing dyes.^115^ Li et al.^116^ fabricated magnetic porous Fe_3_O_4_/carbon octahedra via two-step calcination of Fe-based MOF to eliminate methylene blue. Within 1 h, this material shows 100% removal efficiency in the presence of H_2_O_2_ by a Fenton-like heterogeneous reaction.

### 3.2 Catalytic Properties

Solids with high porosity, large surface area, and high metal ion densities are desirable for catalysis. Numerous MOFs have a surface area of about 5000 m^2^/g or more,^103^ and the presence of metallic nodes brings out the catalytic performance for several reactions. One of the reasons MOFs are more advantageous than other solid catalysts is the probability of structure and pore dimension prediction by knowing the structure of the linker and directionality of coordination among the metal clusters. For example, by replacing one linker with another having increased dimensions but the same geometry of carboxylate units, reticular MOFs can be obtained.^104^ MIL-101(Cr) is frequently used as an acid catalyst in aqueous solutions. It comprises two zeotypic mesopores of 29 and 34 Å diameter and a surface area of 4100 m^2^/g and is highly stable in catalytic reactions.^105^

The clusters of the framework act as nanosized oxides, and the surrounding linkers work as antennae.^106^ MOFs with transition metals such as Co, Cr, and Fe act as oxidation catalysts.^107^ MOF provides different active sites such as metal nodes with exchangeable coordination positions that are unconnected to linkers, modified ligands with attached active sites, or inclusion within the void volume of the active species utilized as oxidation catalysts. Depending on the type of oxidation, these catalysts attain a different level of efficiency.^108^

**Figure 9:**
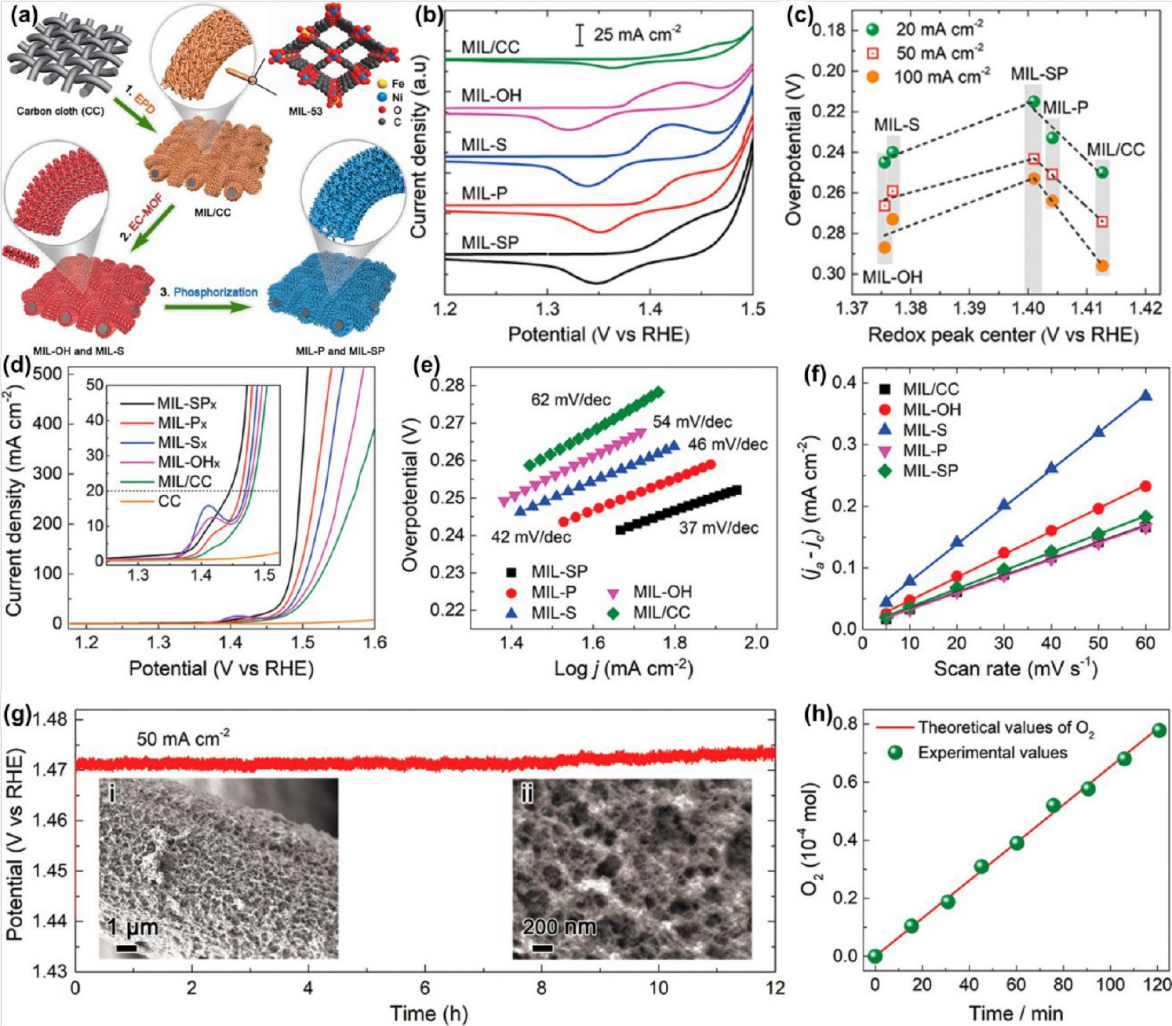
(a) Graphical description of the NiFe-based oxygen-evolving standard materials synthesis method from (Ni, Fe)-MIL-53 predecessors. EPD, electrophoretic deposition; EC-MOF, electrochemical conversion of MOFs. (b) CV curves illustrating the Ni^2+^/Ni^3+^ redox heights for individual materials were documented at a sweep speed of 10 mV s^–1^. (c) The relationship between the weighted center and catalytic performance Ni^2+^/Ni^3+^ redox elevations. (d) OER polarization arcs of the various specimens at a sweep speed of 2 mV s^–1^. (e) Interrelated Tafel slops. (f) At a fixed current density of 10 mA cm^–2^, the time for MIL-SP versus O_2_ measured experimentally (green sphere) as well as theoretically (red solid) for 2 h is described. (g) Solidity trial for 12 h of MIL-SP at a fixed current density of 50 mA cm^–2^. The inset illustrates the SEM pictures of MIL-SP after a 12 h OER process. (h) Capacitive current density distinctions (Δ*j* = *j*_a_ – *j*_c_) are plotted as a part of the sweep speeds. Reprinted with permission from ref (117). Copyright 2019 American Chemical Society.

Wen et al.^117^ proposed the synergistic coupling of anionic ligands to optimize the electronic and catalytic characteristics of MOF-altered oxygen-evolving catalysts. The synthesis of NiFe-doped oxygen evolution reaction (OER) materials with four ligands is shown in Figure 9a. Cyclic voltammogram (CV) graphs attributed to the Ni^2+^/Ni^3+^ couple within Figure 9b show that all materials exhibit two oxidation peaks and one prominent anodic peak (*V*_R_). In Figure 9c, a volcano-shaped arc was developed by *V*_redox_ of the various catalyst scales with their OER performances. IR-corrected OER polarization arcs within Figure 9d indicate that MIL-SP displays the most useful OER performance on overpotentials (η) of just 215, 242, and 253 mV and exhibits valuable current densities of 20, 50, and 100 mA cm^–2^ correspondingly. Further, MIL-SP displays the lower Tafel plot (37 mV/dec, Figure 9e), revealing exceptional OER kinetics. The O_2_ grown from the MIL-SP materials was estimated by amount through a gas chromatography study (Figure 9f). The almost equal portions of experimentally evaluated and theoretically measured O_2_ show an about 100% faradaic efficiency toward the H_2_O-splitting reaction. Furthermore, for 12 h, MIL-SP may maintain its OER performance while on a fixed current of 50 mA cm^–2^. Fascinatingly, MIL-SP morphology is rebuilt during the catalytic process within 3D-linked systems constructed through ca. 10 nm nanoparticles (insets i and ii within Figure 9g). To clarify the outcome of morphological characteristics upon the materials’ OER performance, the ECSAs were estimated through varying sweep rate CV trials (Figure 9h).

## 4 Mechanism of MOF-Based Materials in the Removal of Pollutants from Water

**Figure 10:**
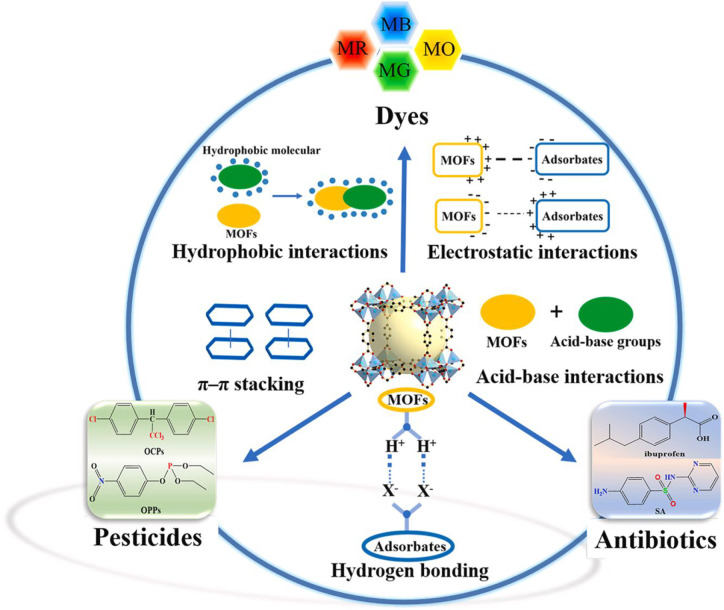
Adsorptive mechanisms of the MOFs. Reprinted with permission from ref (85). Copyright 2021 Elsevier.

The mechanism of the removal of toxic elements utilizing MOFs most commonly uses π–π interactions, ion exchange, H-bonding, acid–base interactions, and electrostatic interactions.^118^ Electrostatic interactions are crucial in adsorption processes between surface charges on pollutants (adsorbates) and oppositely charged MOFs (adsorbents). Net surface charges lead to protonation and deprotonation, which favors the electrostatic interactions between the MOFs and pollutants.^85^ Further, the interactions between hydrogen atoms in N–H, F–H, and O–H bonds and lone pairs of electronegative atoms are known as H-bonding. Studies have revealed that MOFs contain −OH groups and make H-bonds with adsorbates. Acid–base interactions are other mechanisms for the adsorptive removal of MOFs. For instance, Hasan et al.^119^ removed naproxen and clofibric acids from an aqueous solution using MIL-101 functionalized with acidic (−SO_3_H) and basic (−NH_2_) groups. The results revealed that acid–base interactions were dominant and performed better for eliminating acids than did bare MIL-101. Adsorptive mechanisms of MOFs in removing contaminants from wastewater are described in Figure 10.^85^ Relatively higher surface areas of MOFs facilitate the adsorption of pollutants. Adsorption capacity depends on the characteristics of MOFs as well as on the contaminants. The dominant interactions significantly influence the adsorption mechanism. Therefore, the actual adsorption mechanism is complex, and further investigations are required for exact predictions.

**Figure 11:**
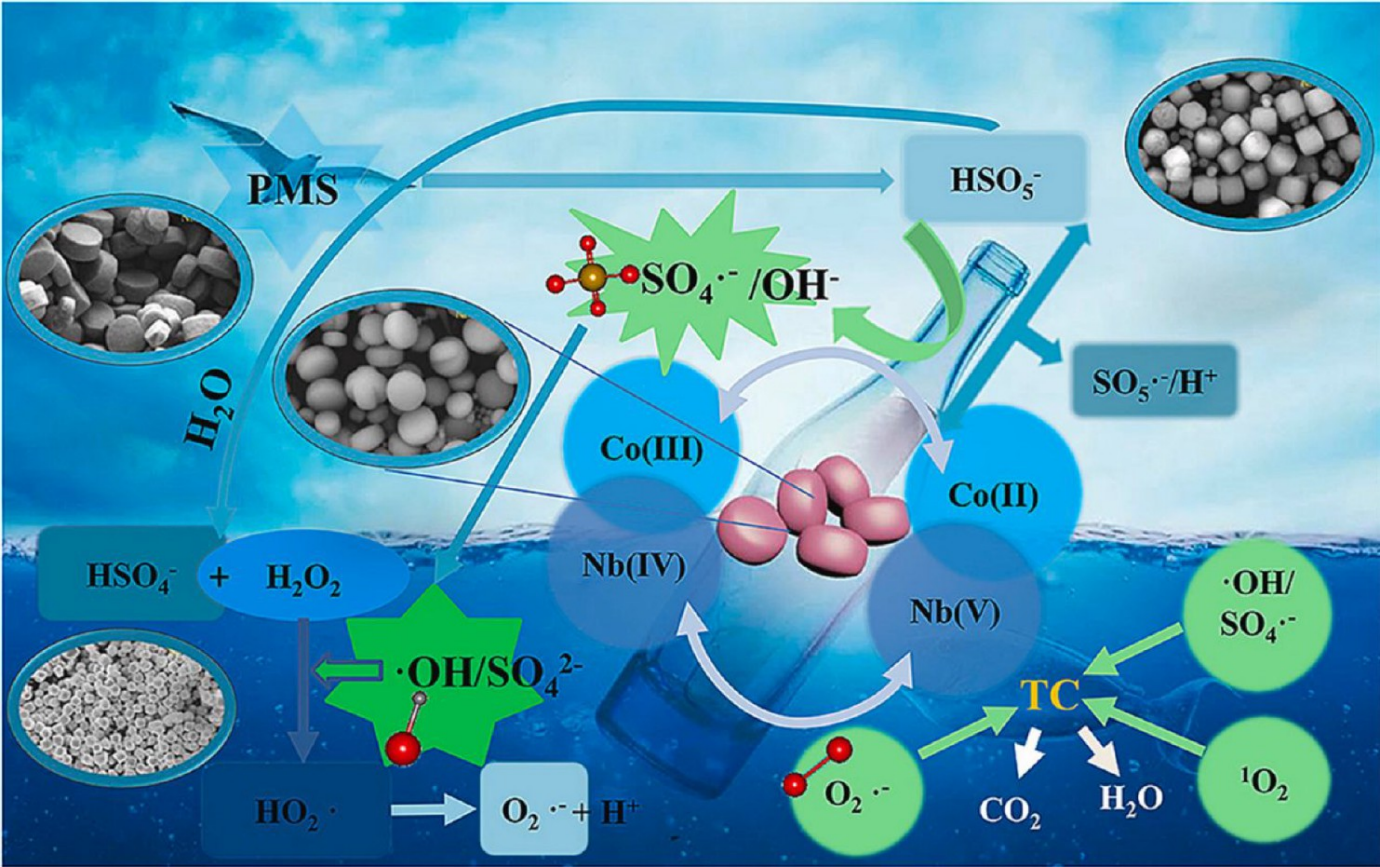
Schematic illustration of the catalytic mechanism of NbCo-MOF. Reprinted with permission from ref (127). Copyright 2022 Elsevier.

In the adsorption mechanism, toxic pollutants only transfer to the surface of MOFs, and their desorption may result in secondary contaminants.^120^ Therefore, for the degradation of pollutants from wastewater, catalysts, oxidizing agents, and some reactants are utilized by sewage treatment plants and factories.^121−123^ Over the past few years, MOFs have attracted much attention for degrading contaminants from wastewater. In catalytic processes, MOFs are combined with highly reactive species such as sulfate radicals (SO_4_^•–^) and hydrogen peroxide (H_2_O_2_) to promote the oxidation of pollutants into less toxic products.^124−126^ NbCo-MOF was synthesized to remove tetracycline from wastewater via SO_4_^•–^ oxidation. The catalytic mechanism of the removal of tetracycline is shown in Figure 11. The observed removal efficiency was 99.7% within 30 min.^127^ Because of the unique structures of MOFs, closer contacts are provided between pollutants and active sites of MOFs, which enhance the reactions between them. MOFs have several advantages, such as high flexibility, highly selective and relatively higher degradation due to rational design, and different pore structures over other catalysts.

## 5 Application of Metal–Organic Framework-Based Materials for Wastewater Treatment

**Figure 12:**
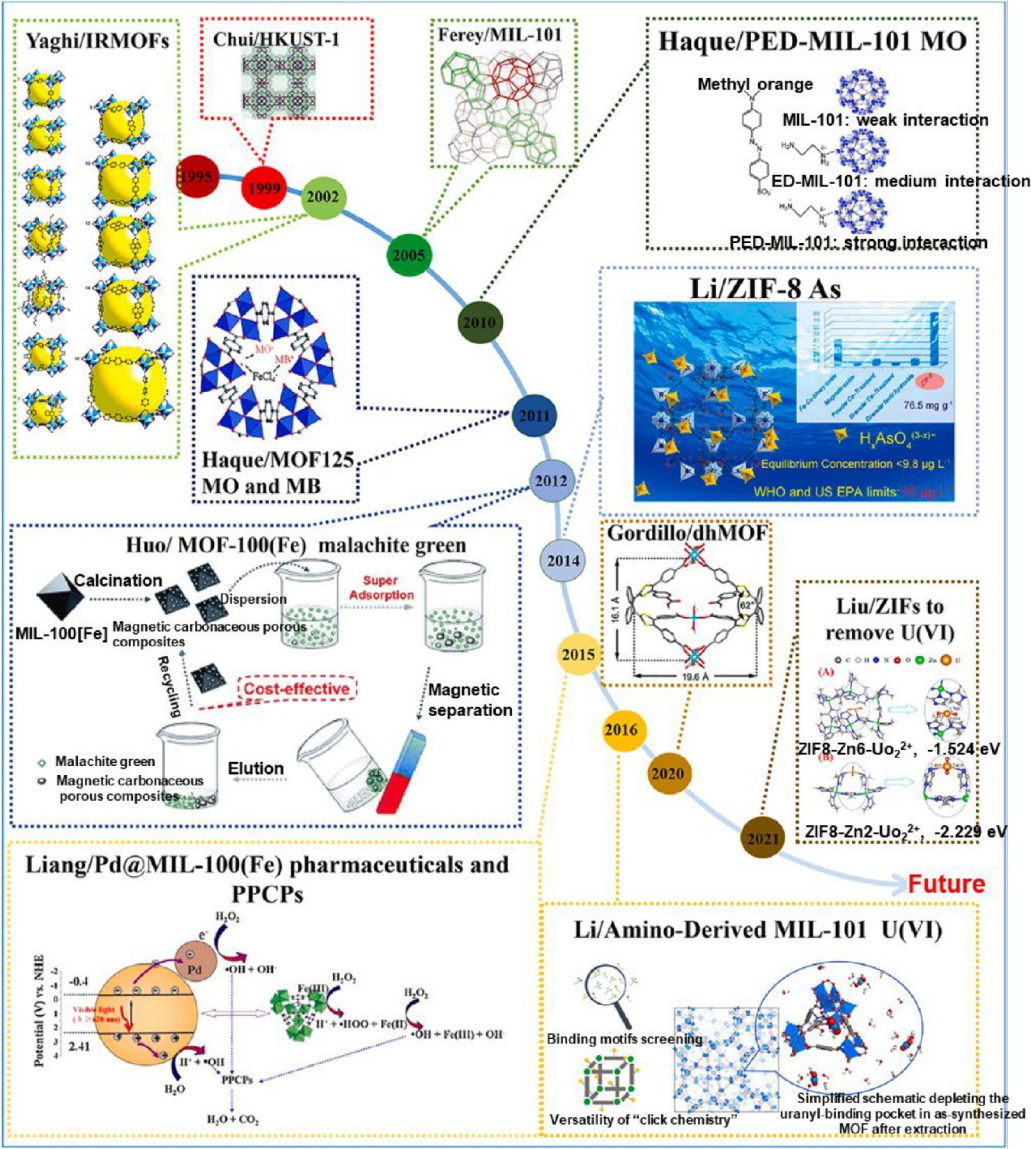
Applications of MOFs toward the elimination of various contaminants from water from the past few years to the present. Reproduced with permission from ref (128). Copyright 2021 Elsevier.

Natural water sources are polluted by many harmful chemicals released from industrial, agricultural, medicinal, and domestic manure that harshly damage the ecological system. MOF-derived materials can be utilized as an adsorbent and catalyst for various processes to eliminate contamination from wastewater due to a higher surface area and tunable pore size. Because of the outstanding separation capability, flexible and controllable size, and composition, MOF-based materials act as scavengers in eliminating heavy metals, organic dyes, pesticides, and other contaminants from wastewater. Therefore, one can say that MOF-based materials are admirable precursors for wastewater treatment in academic research and industrial applications. Figure 12 showcases the applications of MOFs toward the elimination of various contaminants from water from the past few years to the present.^128^ This section provides information regarding the use of MOF-based materials for wastewater treatment.

### 5.1 Removal of Antibiotics

**Figure 13:**
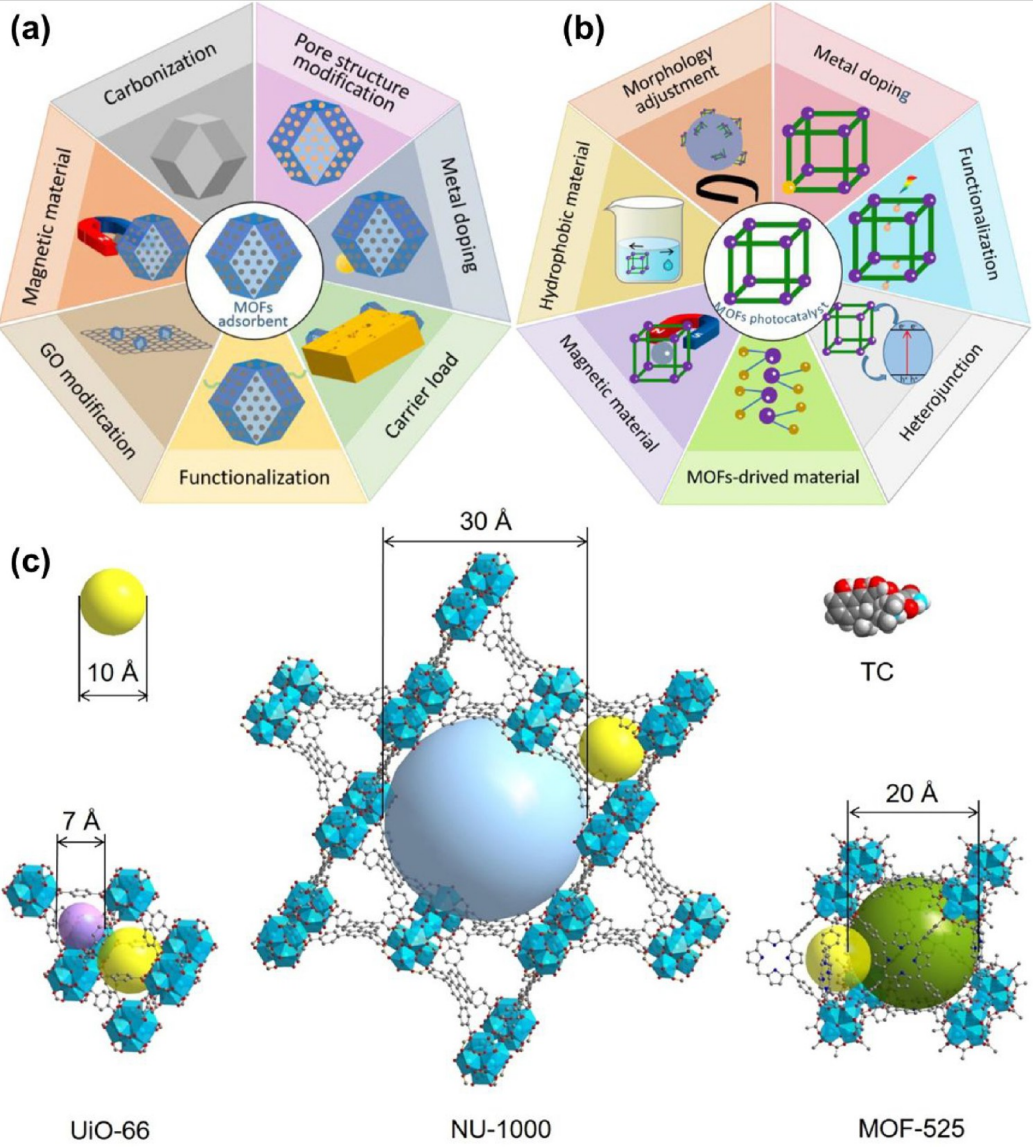
(a) MOF-doped adsorbents. (b) Photocatalysts. Reprinted with permission from ref (131). Copyright 2021 Elsevier. (c) Framework assemblies of MOFs. Reprinted with permission from ref (110). Copyright 2021 Elsevier.

Antibiotics are chemical substances that are generally used in medical treatment and aquaculture. Only 30% of the antibiotic can be absorbed by animals and human beings, and the rest is excreted, say, by urine, which contaminates water sources. In addition, these pharmaceutical industries emit a large amount of antibiotics that severely affect water treatment and are hazardous to the ecosystem.^129,130^ Many MOFs-based materials have been reported for the elimination of antibiotics through adsorption, catalysis, and photodegradation.^131^ Xia et al.^110^ designed Zr-MOFs for the adsorptive removal of tetracycline (TC). Three types of Zr-MOFs were obtained, UiO-66, NU-1000, and MOF-525. For the former two, the adsorption equilibrium was reached within 40 min, while for the latter it was reached within 120 min. The adsorptive capacity of TC on UiO-66 was 145 mg/g, on NU-1000 was 356 mg/g, and on MOF-525 was 807 mg/g. In terms of capacity and adsorption rate, the best performances were given by MOF-525 and NU-1000, respectively. The pore features and topology of MOFs significantly impacted the adsorption execution. The cells whose size matched nicely with TC allowed MOF-525 to obtain the highest adsorption quantity per surface area among the MOFs we inspected. The correct topology of NU-1000 contributed to its high adsorption speed. Figure 13 represents the (a) MOF-based adsorbent, (b) photocatalysts,^131^ and (c) framework structures of MOFs.^110^ Another team of researchers^132^ synthesized MOF-based material Fe_3_(hexaiminotriphenylene)_2_ for the removal of TC. For the synthesis, the team made a solution of 25 mL of distilled water with 0.36 mmol of FeCl_3_ and added this solution at room temperature to another solution containing water and 2,3,6,7,10,11-hexaiminotriphenylene (HITP)·6HCl. Further, 14 M ammonia solution was added. Dark solid precipitates were obtained, which were continuously stirred for 3–4 h. The supernatant then was removed, and the remaining solid was stirred with acetone and water for 24 h. Finally, centrifugation of the resulting powder was done and dried at 60 °C in a vacuum. The authors observed that the MOF-based material showed a removal percentage of 62.7% within 30 min and showed excellent catalytic performance of 97.7%.

**Figure 14:**
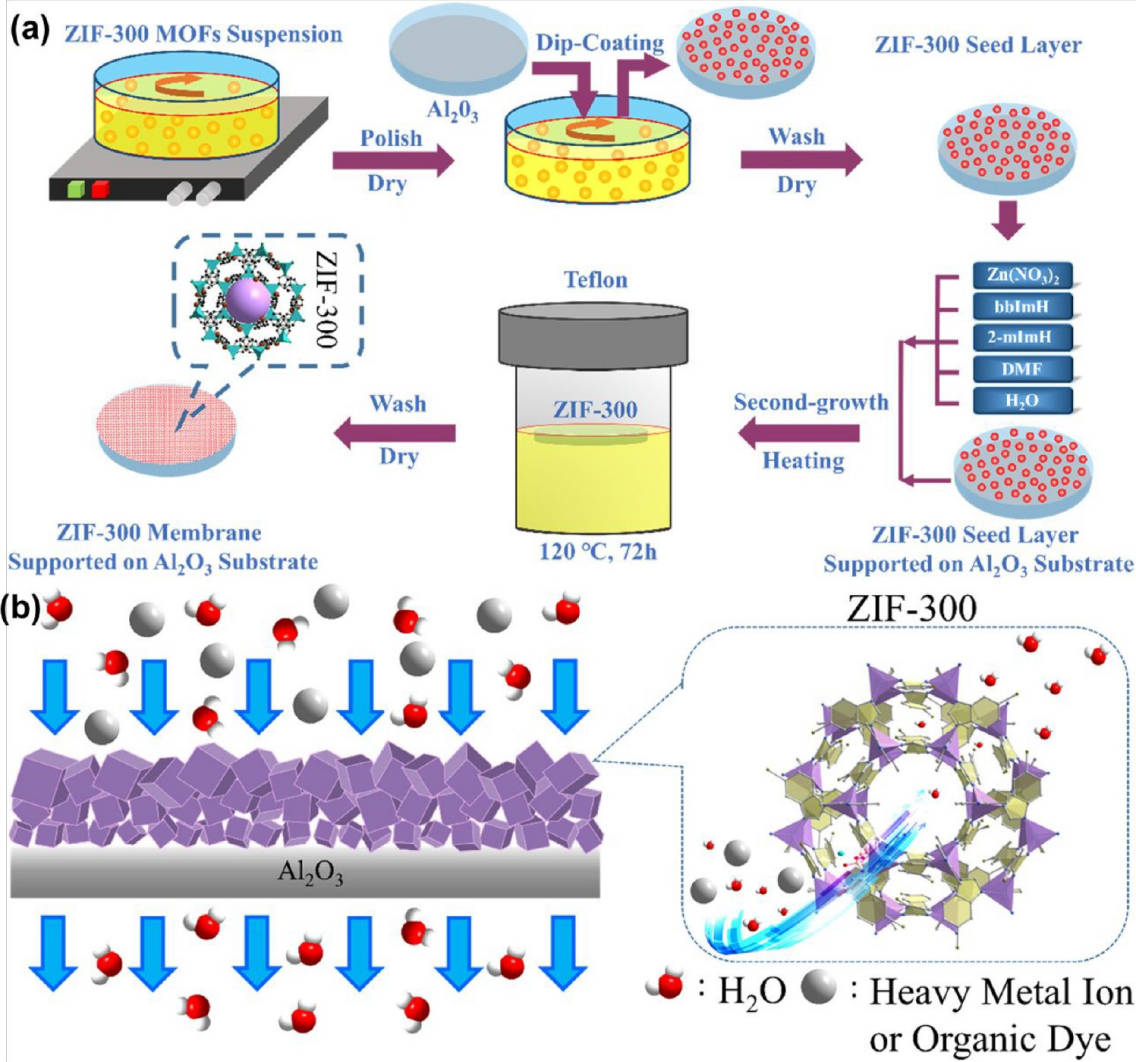
(a) Systematic synthesis procedure of the ZIF-300 membrane. (b) Elimination process of heavy metals via the size exclusion mechanism. Reprinted with permission from ref (134). Copyright 2019 Elsevier.

For the elimination of quinolones in wastewater, bimetallic magnetic Fe_*x*_Mn_*y*_ catalysts were synthesized by Li et al.^133^ via the impregnation method. The formed MOF-based material exhibits good porous structure with a high surface area of 122.5 m^2^/g. Researchers observed that, within 30 min without utilizing any kind of oxidant, the material can degrade 98.3%, 96.0%, 91.0%, 92.2%, and 93.5% of ciprofloxacin (CIP), ofloxacin (OFL), enrofloxacin (ENR), levofloxacin (LEV), and norfloxacin (NOR), respectively. Figure 14 represents (a) the systematic synthesis procedure of the ZIF-300 membrane and (b) the elimination process of heavy metals via the size exclusion mechanism.^134^ The fabricated membrane exhibits a 99.21% rejection rate and a water permeance of 39.2 L/m^2^·h·bar.

Table 2 showcases different MOFs-based materials utilized for the effective elimination of antibiotics from wastewater.

**Table 2:** Different MOFs-Based Materials Utilized for the Elimination of Antibiotics from Wastewater^a^

MOF-based material	pollutant removed	preparation method	adsorbent/catalyst dosage (g/L)	pH	surface area (m^2^/g)	pore size (nm)	removal efficiency (%)	performance	ref
CFC/UiO-66-NH_2_/AgI	CIP	solvothermal-chemical sedimentation	10 mg/L	4.5–8.5	730.8	2.78	79.6	shows a broader absorption edge (∼440 nm)	(135)
	LEV						84.5		
Fe_*x*_Mn_*y*_	quinolones	facile impregnation method	0.007		122.5	13.7	92	degrades quinolones in 30 min without any oxidant	(133)
copper *meso*-tetra(4-carboxyphenyl) porphine-MOFs	oxytocin	microwave-assisted hydrothermal method	0.2	5	342.72	0.256	83.6	displays adsorption capacities of 130, 150, and 50 mg/g for oxytocin, TC, and NOR, respectively	(136)
	TC			95					
	NOR								
MIL-101	TC	solvothermal method	0.15	10.2	180.41	7.55	82.52	shows pore volume of 0.32 cm^3^/g	(137)
UiO-66-NH_2_	NOR	hydrothermal route	0.1	8	713.2	12	91.6	highest partition coefficient of 20.9 mg/g/μM was observed	(138)
Zn_3_(BTC)_2_	OFL	sonochemical method	10 mg/L	6.7			72	displays adsorption capacity of 25.3 ± 0.8 mg g^–1^	(139)
Zr/Fe-MOF/GO	TC hydrochloride		20 mg/L	1–14			97.8	it follows pseudo first- and second-order kinetics	(140)
alginate-graphene-ZIF67	TC	in situ route	1	4	138.62	15.43	99.73	displays adsorption capacities of 456.62 mg/g	(141)
UiO-66-(COOH)_2_/GO	TC	reflux heating method	0.5	3	369.6	5.04		have quantum efficiency of 164.91 mg/g	(142)
UiO-66-NH-BT@g-C_3_N_4_	sulfamethoxazole	step-by-step in situ growth strategy		5		40	97.6	initial concentration of antibiotic was 10 mg/L	(143)

a CFC/UiO-66-NH_2_/AgI, carbon fiber cloth/amine-functionalized zirconium metal organic framework/silver iodide; Zn_3_(BTC)_2_, Zn(II) and benzene-1,3,5-tricarboxylate.

### 5.2 Removal of Organic Dyes

Dyes, when mixed with water, cause adverse effects on marine life, animals, and human beings. Dyes hardly degrade due to their xenobiotic characteristics and complex structure. To dye cotton and wood, methylene blue has been extensively used, which produced a large amount of colored wastewater.^144^ Globally, around 10 000 tons of dyes is used by textile industries annually.^145,146^ Dyes generally have high color intensity and carcinogenic and mutagenic properties that result in the toxicity of the effluents. Dyes have detrimental effects on the renal system, respiratory system, eyes, skin, and liver of humans. Acidic dyes can even cause cancer.

Further, organic dyes have toxic effects on aquaculture. Therefore, the elimination of dyes from wastewater is essential.^147,148^ Numerous methods have been developed to eliminate dyes from water sources such as adsorption, coagulation, oxidation, and biological degradation. MOF and MOF-based materials have a high potential to remove dyes from wastewater by adsorption and catalysis. Dyes are generally cationic or anionic in nature; examples of former dyes are methylene blue (MB) and rhodamine B (RhB), while Congo red and methyl orange are examples of the latter. MOFs also possess either a positive charge or a negative charge. Positively charged MOFs attract negatively charged dyes, and negatively charged MOFs attract cationic dyes.^149^ These electrostatic attractions are the driving force for the adsorption mechanism of MOFs toward dyes. Hydrogen bonding, π–π interactions, and physical adsorption are other mechanisms of adsorption of dyes on MOFs. The adsorption mechanisms of dyes on MOF surfaces typically depend on many factors.

#### 5.2.1 Effect of Pore Size

The pore size and geometry of MOF-based nanomaterials are crucial for the adsorptive removal of dyes. Because of a higher surface area, porous MOFs can absorb greater quantities of dyes than can nonporous MOFs. A flexible or adjustable design results in several MOF frameworks with multiple pore sizes, dimensions, and geometries. For instance, MOFs such as IRMOF-01 and PCN-222 have square grid channels; PCN-224 and MOF-74 have hexagonal channels.^111,112^ If the pore size of MOF-based nanocomposites is larger than the size of the dye molecules, then a considerable amount of dye can be adsorbed on the surface of the MOF. However, if the size of the molecules of dyes is larger as compared to the MOF frameworks, then the adsorption of dye molecules is ruled out within the pores of the MOF.^113^

#### 5.2.2 Effect of Functional Groups

The adherence of adsorbates is promoted by particular functional groups present on MOF-based nanomaterials such as amino groups interacting with acidic dyes and sulfonic acid interacting with basic dyes and with pore geometries of porous MOFs.^99^

Dyes can facilely be degraded via the oxidation process. The Fenton advanced oxidation process is considered a potential method for removing dyes.^115^ Li et al.^116^ fabricated magnetic porous Fe_3_O_4_/carbon octahedra via two-step calcination of Fe-based MOF for the elimination of methylene blue. Within 1 h, this material shows 100% removal efficiency in the presence of H_2_O_2_ by a Fenton-like heterogeneous reaction. Table 3 showcases different MOFs-based materials utilized to degrade dyes from wastewater.

**Table 3:** Different MOFs-Based Materials Utilized for the Elimination of Dyes from Wastewater

MOF-based materials	dyes removed	preparation method	initial concentration of dye (mg/L)	surface area (m^2^/g)	pore size (nm)	removal rate	pH	performance	ref
Ce(III)-doped UiO-67 nanoparticles	Congo red	solvothermal method	>100	1911.9	80			displays adsorption capacity of 799.6 mg/g	(150)
Fe-MIL-88NH_2_	Congo red	solvothermal method	5–60			87.2%		reached the equilibrium in 60 min	(151)
MOF@Ox-cotton hybrids	MB	infrared assisted method	50		55–125			shows adsorption capacity of 75.46–187.03 mg/g	(152)
	RhB								
Co-MOF	methyl orange	solvothermal method	30 ppm			79.56%	4–5	displays adsorption capacity of 18.80 mg/g and 4.57 for reactive black and methyl orange, respectively	(153)
	reactive black 5								
Ce-MOF@Fe_3_O_4_@activated carbon	indigo carmine and methylene blue	coprecipitation method	10			99%	7	shows maximum adsorption capacity of 85.5 and 84.9 mg/g for indigo carmine and methylene blue	(154)
Sm-MOF/GO	RhB	in situ method	10			91%		rejection rate was maintained even after continuous 5.5 h filtration	(155)
Cu-MOFs/Fe_3_O_4_	malachite green	in situ method		35.4	3.5	90%		displays adsorption capacity of 113.76 mg/g	(156)
MOF/porous carbon	MB	one-step carbonization treatment	200	1338	3.2			shows adsorption capacity of 2724 mg/g	(157)
Zr-sulfonic @MOF	MB	solvothermal method	20			93%	7	shows maximum adsorption capacity of 1992 mg/g	(158)
MIL101-Cr/PANI/Ag	MB	hydrothermal method	25	2861	153	97%	12	displays adsorption capacity of 43.29 mg/g	(159)

### 5.3 Removal of Heavy Metals

Heavy metals are detected in traces and have detrimental effects on aquatic life. Commonly present heavy metals in wastewater are Cu, Cd, Zn, Hg, Pb, Ca, and Co. Corroded plumbing systems and cable industries are the major sources of Cu in wastewater. Brass coatings and aerosol deodorants are the sources of Zn contamination, while batteries and alloys are the main sources of Pb.^160,161^Table 4 showcases different MOFs-based materials used for the elimination of heavy metals from wastewater. An ethylenediamine-functionalized Zr-based MOF was synthesized to effectively absorb heavy metals from wastewater.^162^ The composite was prepared through the Michael addition reaction and has an adsorption capacity of 243.90 mg/g for Pb^2+^, 208.33 for Cu^2+^, and 217.39 for Cd^2+^. Zeolite imidazolate framework-300 was synthesized for the potential elimination of heavy metals from wastewater.^134^ The authors synthesized the MOF via the second-growth method and observed a rejection rate (99.2%) for CuSO_4_ and high water permeance of 39.2 L/m^2^h·bar. For the adsorptive removal of Pb (II), Shi et al.^156^ fabricated CuMOFs/Fe_3_O_4_ via doping of Fe_3_O_4_ nanoparticles on the in situ growth of Cu-MOFs and showed an adsorption capacity of 219 mg/g. The surface of CuMOFs/Fe_3_O_4_ was 35.4 m^2^/g and had a removal efficiency of 96%. Chitosan-MOF composite was prepared for the potential degradation of Cr^2+^, Cu^2+^, and Ni^2+^ from wastewater and at 40 °C had 93.6 mg/g adsorption capacity at pH 2 for Cr (VI), while for Cu^2+^ and Ni^2+^ the adsorption capacities at pH 5 were 50.6 and 60 mg/g at 60 and 20 °C, respectively.^163^ Zayan et al.^164^ synthesized polypyrrole/aluminum fumarate-MOF by in situ oxidative polymerizations for the effective elimination of Pb from the wastewater stream. The prepared composite had a larger surface area of 809 m^2^/g and exhibits a removal efficiency of nearly 100% in the pH range from 3 to 7.

**Table 4:** Different MOFs-Based Materials Used for the Elimination of Heavy Metals from Wastewater

MOF-based materials	pollutants removed	adsorbent dosage (g/L)	pH	surface area (m^2^/g)	adsorption capacity (mg/g)	kinetic mode	preparation method	ref
Fe/Mg-MIL-88B	As (V)		7	360	303.6	pseudo-second	hydrothermal	(165)
2D-ZIF-L	As (III)	0.1	10	67.02	43.74	pseudo-second		(166)
γ-cyclodextrin MOF-based nanoporous carbon	Cd (II)		7	11.4	140.85	pseudo-second	carbonization	(167)
Fe-gallic acid MOFs	Cr (VI)	1		297.8	1709.2	pseudo-second	hydrothermal	(168)
ZIF-8	Cr (VI)	0.020	7	1281	0.15		green method	(169)
MOF-808-EDTA	Hg (II)	1		1173	592	pseudo-second	solvent-assistant linker exchange	(170)
	La (III)				205			
	Pb (II)				313			
MOF-545	Pb (II)		7	2129	73	pseudo-second		(171)
Cu-MOFs/Fe_3_O_4_	Pb (II)	10		35.4	219	pseudo-second	in situ	(156)
cadmium terephthalate-based MOF	Pb (II)	1.0 g	5–6		434.78	pseudo-second	ultrasonic	(172)
	Cu (II)				769.23			
mercaptosuccinic@ MOF	Hg (II)	0.1	4		1180	pseudo-second	one-step synthesis	(173)
	Pb (II)				510			
MOF-based	Cr (VI)	0.005	7	975	91%		Pickering emulsion	(174)
Fe-UiOsomes-Pt motors								

### 5.4 Removal of Agricultural Pollutants

Because of the surging demand for food globally, these days, pesticides, fertilizers, and herbicides are widely used for the protection and growth of crops to meet the growing demand. Various agricultural chemicals such as β-lactams, organophosphates, and sulphonamide have been found in wastewater effluents and livestock farms. Worldwide, approximately three million tons of pesticides are used in agricultural land annually, nearly 15–20 times higher than in the past 30 years.^175,176^ These agricultural chemicals have detrimental effects on human health as they can damage the endocrine and nervous systems and can cause irritation and carcinogenic effects.^177,178^

For the photocatalytic degradation of atrazine (ATZ), TC, and sulfamethazine (SMT), MOF-2/graphitic carbon nitride (g-C_3_N_4_) nanosheets were prepared by Wang et al.,^179^ by the vacuum-assisted self-assembly method. MOF-2 nanosheets were synthesized by utilizing top-down delamination of bulk MOF-2, while g-C_3_N_4_ nanosheets were synthesized through the chemical exfoliation of bulk g-C_3_N_4_. The as-prepared nanosheets exhibit a maximum removal efficiency of 98%, 95%, and 89% for ATZ, TC, and SMT, respectively, at a permeable flux of 23.6 L m^–1^ h^–1^ bar^–1^. For the adsorptive elimination of imidacloprid from water, calcium fumarate-MOF was developed,^180^ which shows an adsorption capacity of 476.23 mg/g at pH 6.5 with a 98% removal rate within 70 min.

**Figure 15:**
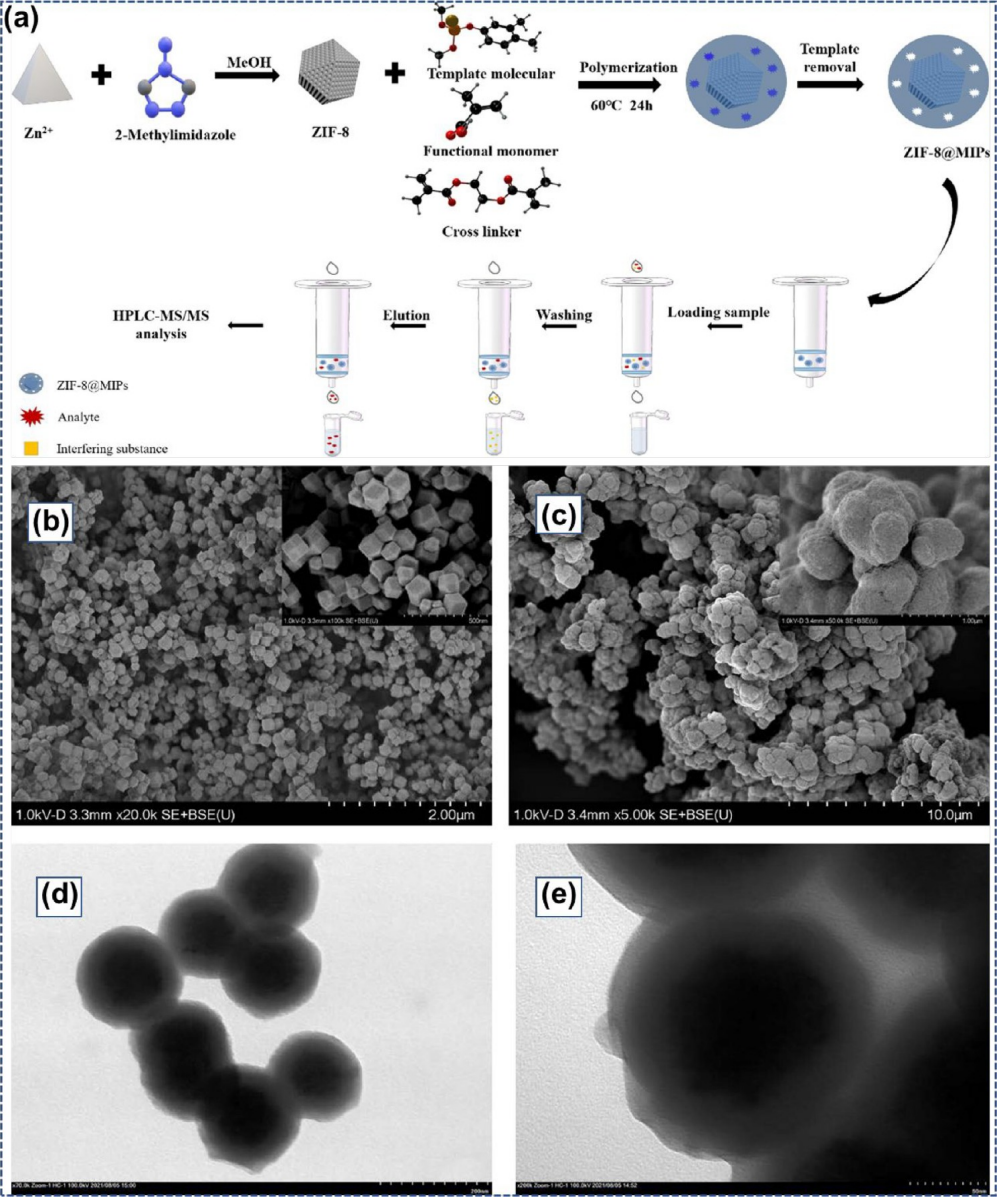
(a) Graphic illustrations of ZIF-8@MIPs synthesis and the SPE method. SEM pictures of the ZIF-8 (b) and ZIF-8@MIPs compounds (c). TEM pictures of ZIF-8@MIPs (d,e). Reprinted with permission from ref (181). Copyright 2022 Elsevier.

Yang et al.^181^ synthesized MOF @ molecularly imprinted polymer (ZIF-8@MIPs) adsorbents for the solid-phase extraction of organophosphorus pesticides by agricultural derivatives. Figure 15a shows the graphical illustration for preparing ZIF-8@MIPs via the solid-phase extraction (SPE) method. SEM and TEM characterized the morphological arrangement of the synthesized ZIF-8 and ZIF-8@MIPs. As demonstrated in Figure 15b, ZIF-8 exhibited smooth facades and rhombic dodecahedral shapes with intense sharpness. 100–200 nm was the observed diameter. Corresponding with pristine ZIF-8, the exterior of the mixed composites became brutish with noticeable folds. Successful polymerization of the homogeneous imprinted coating upon the exterior of ZIF-8 was confirmed by the more extensive size of the ZIF-8@MIPs materials (Figure 15c). Also, as shown by typical TEM pictures (Figure 15d and e), the consistency of the MIPs shell with an average of approximately 50 nm was uniformly polymerized on the exterior of the ZIF-8 core to deliver a generally core–shell arrangement. Another organophosphorus pesticide, glyphosate, was removed by the MOF-based material Fe_3_O_4_@SiO_2_@UiO-67.^182^ The material was synthesized through layer-by-layer assembly, and the material consists of Zr–OH groups that have a high affinity for phosphate-containing groups that enhance the adsorption capacity of the material. The material achieved an adsorption equilibrium within 60 min and exhibited an adsorption capacity of 256.54 mg/g with a lower limit of detection (0.093 mg L^–1^). Jia et al.^183^ synthesized MOF-hydrogels (ZIF-8 on Zn2@ sodium alginate) via the layer pillar strategy. This system successfully detected thiophanate-methyl pesticide in real vegetables and fruits within a wide linear range from 10 × 10^–6^ to 100 × 10^–6^ in a low detection limit of 0.14 × 10^–6^. When thiophanate-methyl was sprayed on fruits and vegetables, it produced carbendazim, which was also removed by the prepared material and exhibits an adsorption capacity of 161.8 mg/g. The sensor had a good recyclability with recovery rates in the range of 98.3–102.7%.

In addition to this, a cationic MOFs-based sensor was developed by Wu et al.^184^ for the detection of six phenoxy carboxylic acid herbicides from water samples. The material was synthesized by soaking Zr-MOFs in the solution of polyvinylidene fluoride followed by functionalization with quaternary amines. The adsorption performance was enhanced by π–π conjugation and cation−π bonding within the detection limit of 0.03–0.059 ng/L and had good recovery rates (80–117%). To remove the insecticide named imidacloprid from water, a team of researchers developed a sensor by utilizing polyethylene terephthalate as a source for the preparation of UiO-66 frameworks.^185^ The adsorption equilibrium was reached within 60 min, and the sensor exhibits good stability and recyclability. The obtained adsorption capacity was 467.23 mg/g. For the removal of triazole, Cu-based MOF was fabricated by utilizing the Fe_3_O_4_-graphene oxide (GO) nanocomposite from the water sample.^186^ The material exhibits a correlation coefficient of 0.992 and a detection limit of 0.05–0.1.

## 6 Challenge to Wastewater Treatment and Role of MOFs

For the removal of toxic materials from wastewater resources, numerous techniques such as ion exchange,^187^ solid-phase extraction,^188^ electrochemical-based methods,^189^ and precipitation^190^ have been utilized over the past couple of years. However, these methods have several demerits, such as complex removal processes and special working conditions. However, due to their simple and straightforward design, user-friendly and cost-effective adsorption techniques are considered an alternative for eliminating contaminants.^175,191−194^ Many materials, such as activated carbon^195^ and clay–polymer composites,^196^ have been extensively utilized as adsorbents to remove toxic particles from water resources. However, limitations such as lower adsorption capacities and tedious separation process limit their effectiveness.^197^

Because of their attractive properties, such as large surface area and pores and high chemical and solvent stability, MOFs have been explored to remove hazardous particles from wastewater.^198,199^ Although for widespread applications of MOFs for removing toxic materials, several limitations, such as a more straightforward separable design, have to be overcome. For instance, Abdel-Magied et al.^193^ synthesized Fe_3_O_4_@UiO-66-NH_2_ by ultrasonication to remove Cd^2+^ and Pb^2+^ from an aqueous solution. The obtained maximum adsorption capacity was 714.3 and 833.3 mg/g for Cd^2+^ and Pb^2+^, respectively, with excellent reusability.

Kavand et al.^200^ conducted a study by utilizing activated carbon for the removal of Cd^2+^ and Pb^2+^ from wastewater. The adsorption capacities for the former and latter ions were only 9.26 and 9.30 mg/g, respectively. The polymer–clay-based composite was synthesized to remove Pb^2+^ from the aqueous solution. The maximum adsorption capacity was 21 ± 0.39 mg/g.^201^ Further, attapulgite clay@carbon was fabricated by Chen et al.^202^ for the removal of Pb^2+^ by a one-pot hydrothermal carbonization process. The authors observed a maximum adsorption capacity of 263.83 mg/g. These examples show that the adsorption capacity of MOFs for the removal of heavy metal ions is more than that of other materials. SO_3_H-UiO-66 (18%) was fabricated by Hasan et al.^203^ for the adsorptive removal of diclofenac. The authors observed that the adsorption capacity of the as-synthesized material was 263 mg/g, nearly 13 times higher than that of conventional activated carbon (76 mg/g) under similar conditions. The surface area for the MOF-based material was 910 m^2^/g. A MOF (MIL-53 (Cr)) was fabricated by a team of researchers^204^ for the effective removal of 2,4-dichlorophenoxyacetic acid from contaminated water. The adsorption capacity of MIL-53 was 556, which is much higher, nearly twice that of activated carbon and zeolite. It removed MB and methyl orange from polluted water by utilizing the adsorption property of MOF-235.^2^ The authors compared the performance of MOF-235 with that of conventional activated carbon. The adsorption capacity of MOF-235 was 477 and 187 mg/g for methyl orange and MB, respectively, while the adsorption capacity of activated carbon for the former dye was only 11.2 mg/g, and for the latter dye it was 26 mg/g.

## 7 Advantages of MOF-Based Materials over Other Materials

MOFs can be one of the best supporting active species because they can prevent leaching of homogeneous catalyst either by encapsulating the cavities or by forming covalent bonds with the framework.^205^

Zeolite-type materials need inorganic or organic templates for their formation, while MOFs do not need these external templates as the solvent itself acts as a template for the formation of MOFs.^206^

Most metal cations are utilized in the preparation of MOFs, whereas for the formation of other adsorbents, a few cations (Si, Al, and P) can participate in their formation processes.^207^

Identical ligands and numerous analogues of MOFs can be formed by utilizing different metallic components.^208^

Isoreticular MOFs can be prepared with the same metal species just by changing the length of the ligands.^104^

They are highly specific due to easy modification of the pore sizes and surfaces.^207^

## 8 Conclusion and Future Prospects

We delivered an inclusive review of recent progress in MOF-based materials for the removal of hazardous pollutants. Because of their different effects and possible applications, MOFs may be valuable to materials for removing contaminants from aqueous media. This Review examined recent investigations on the adsorptive reduction of other pesticides, especially from an aqueous stage employing MOF-based materials. This Review could help scientists to comprehend the existing research scenario on pesticide reduction utilizing MOF-based adsorbents. As summarized, few of the MOF-based adsorbents exhibited performance considerably better than that of traditional adsorbents within the adsorption of different pesticides.

The quest for advanced substances with appropriate characteristics has evolved into a unique approach to reduce the ever-increasing concerns associated with environmental decay. We conclude that the significant adsorption capabilities of MOFs primarily resulted from relations among mark ions and active binding clusters upon the MOFs, jointly with the favorably rated porous network that may be restrained to ease the dispersal of the metal ions. Instructing suitable functional sets within MOFs and adjusting their porosity or incorporating the pores of MOFs with isoreticular or postsynthetic methods have been demonstrated to be efficient techniques for improving their selectivity and adsorption capability for poisonous/radioactive metal ions. MOF-based combinations with universal arrangements, surfaces, and effects have been developed successfully and used in broad areas. Specifically, nanostructured composites from MOFs are nominees for environmental cleaning and monitoring due to their superior preparation and execution.

In the coming days, multiple problems must be examined at the laboratory scale, mainly based on acquiring essential learning of adsorption/catalytic/sensing tools and the affinity between the configuration of MOF byproducts. For practical applications of these nanomaterials in the treatment and management of wastewater, ecofriendly and cost-effective methods should be adopted.

Investigators must concentrate on simplifying preparation methods and optimizing costs while seeking routes to improve resilience, selectivity, and reusability. Thus, attention must be paid to fabricating MOF byproducts with outstanding characteristics to maximize their efficiencies and secure industrial implementations in a challenging environment.

There is still a long path to go in applying MOF materials toward (i) large-scale water processing due to its price; (ii) exploring water-stable MOFs for potential functional applications; (iii) investigations on the consequences of radiation on the strength of MOFs have been lacking, and more details could be alluring; (iv) multiple MOFs emanated from costly ligands, and, therefore, more economical options are of significant need; and (v) the long-term durability of MOFs and resurrection designate a challenge concerning secondary pollution and useful applications. Thus, MOFs resistant to structural degradation caused by moistness, oxidants/reductants, acids/bases, and radiation are selected for prospective investigation. Still, future investigation endeavors are anticipated to promote the possibilities of MOF for practical application.

Finally, MOF-based materials, without any suspicion, have appeared as thrilling advanced composites in environment-based areas, where possibilities and challenges coexist. With maintained steps dedicated to this subject, there is an abundance of room to acquire the actual industrial implementation of MOF-derived materials in the area of eco-friendly remediation and monitoring.

## Abbreviations

MOFs: metal–organic frameworks
NMR: nuclear magnetic resonance
DMF: dimethylformamide
STLHC: sertraline hydrochloride
SNDGr: sulfur and nitrogen codoped graphene
ECSA: electrochemical active surface area
CV: cyclic voltammetry
PGE: pencil graphite electrode
EIS: electrochemical impedance spectroscopy
LMOF: luminescent MOF
FTO: fluorine-doped tin dioxide
AC: activated carbon
DBA: 4-(dodecyloxy)benzoic acid
SEM: scanning electron microscopy
spng: sponge-like
pmg: pomegranate-like
OER: oxygen evolution reaction
EPD: electrophoretic deposition
EC-MOF: electrochemical conversion of metal–organic framework
TC: tertracyline
CIP: ciprofloxacin
OFL: ofloxacin
ENR: enrofloxacin
LEV: levofloxacin
NOR: norfloxacin
CFC: carbon fiber cloth
BTC: benzene-1,3,5-tricarboxylate
MB: methylene blue
RhB: rhodamine B
ATZ: atrazine
SMT: sulfamethazine
g-C_3_N_4_: graphitic carbon nitride
MIP: molecularly imprinted polymer
SPE: solid-phase extraction
TEM: transmission electron microscopy
